# Recombinant humanized collagen remodels endometrial immune microenvironment of chronic endometritis through macrophage immunomodulation

**DOI:** 10.1093/rb/rbad033

**Published:** 2023-04-03

**Authors:** Shuang You, Yun Zhu, Hu Li, Fan He, Shuaibin Liu, Xia Yang, Li Wang, Hui Zeng, Jingcong Dai, Lina Hu

**Affiliations:** Department of Obstetrics and Gynecology, The Second Affiliated Hospital, Chongqing Medical University, Chongqing 400010, China; National Laboratory of Biomacromolecules, Institute of Biophysics, Chinese Academy of Sciences, Beijing 100101, China; Department of Obstetrics and Gynecology, The Second Affiliated Hospital, Chongqing Medical University, Chongqing 400010, China; Department of Obstetrics and Gynecology, The Second Affiliated Hospital, Chongqing Medical University, Chongqing 400010, China; Joint International Research Lab for Reproduction and Development, Ministry of Education, Chongqing 400010, China; Department of Obstetrics and Gynecology, The Second Affiliated Hospital, Chongqing Medical University, Chongqing 400010, China; Shanxi Jinbo Pharmaceutical Co., Ltd, Taiyuan, Shanxi 030031, China; Department of Obstetrics and Gynecology, The Second Affiliated Hospital, Chongqing Medical University, Chongqing 400010, China; Department of Obstetrics and Gynecology, The Second Affiliated Hospital, Chongqing Medical University, Chongqing 400010, China; Department of Obstetrics and Gynecology, The Second Affiliated Hospital, Chongqing Medical University, Chongqing 400010, China; Department of Obstetrics and Gynecology, The Second Affiliated Hospital, Chongqing Medical University, Chongqing 400010, China; Joint International Research Lab for Reproduction and Development, Ministry of Education, Chongqing 400010, China; Reproduction and Stem Cell Therapy Research Center of Chongqing, Chongqing 400010, China; Center for Collagen Transformation of Chongqing Medical University, Chongqing 400010, China

**Keywords:** chronic endometritis, recombinant humanized collagen, type III collagen, macrophages

## Abstract

Recently, evidence has suggested that chronic endometritis (CE) is a crucial factor associated with infertility and failure of assisted reproductive techniques, prompting concern in the reproductive field. Studies have shown that persistent infiltered immune cells stimulation result in the disturbance of endometrial immune microenvironment could lead to the infertility of CE patients finally. Conventional treatments are limited because they lack immune regulation, so it is urgent to develop a novel approach to treat CE and promote embryo implantation in patients with CE. Herein, we prepared recombinant humanized type III collagen (rhCol III) with high cell adhesion activity to regulate macrophages and repair the endometrium. In this study, M1 macrophages and M1 macrophages cultured medium and lipopolysaccharide (LPS) co-stimulated inflammatory endometrium stromal cells (ESCs) were established *in vitro* to mimic CE condition. rhCol III promoted M1 macrophages toward M2 phenotype, improved cell migration, viability and collagen components of inflammatory ESCs. Also, the inflammatory response of inflammatory ESCs was downregulated after rhCol III treatment. Subsequently, LPS was used for CE rat model and a 28-day observation was performed; inflammatory cells’ infiltration, endometrium repair, extracellular matrix (ECM) remodeling and pregnancy outcomes were promoted after rhCol III endometrial infusion. In conclusion, rhCol III promoted (i) macrophage polarization toward M2 macrophages, (ii) pro-inflammatory cytokine production and anti-inflammatory cytokine reduction, (iii) ECM remodeling and (iv) fertility restoration. Meanwhile, rhCol III enhanced cell biological functions by interacting with discoidin domain receptors, regulated cell metabolism and reduced the inflammatory response through the inhibition of the NF-κB/YAP signaling pathway. Overall, the results illustrated the potential therapeutic prospects of rhCol III for CE treatment.

## Graphical abstract

**Figure:**
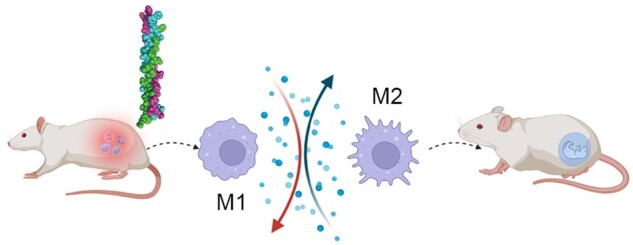

## Introduction

With the high prevalence of infertility worldwide and the development of assisted reproductive techniques, there has been an increase in research focused on chronic endometritis (CE) and infertility. Some researches indicate that CE might lead to unexplained infertility and poor success rate of *in vitro* fertilization [1–3]. According to some publications, women with CE had higher infertility risk compared with women without CE, including recurrent miscarriage (27–57.8%) and repeated implantation failure (15–42%) [4–8].

CE is a persistent inflammation and immune responses of the endometrial mucosa. It is characterized by the presence of edema and massive immune cell infiltration in the stroma, which results in an aberrant endometrial microenvironment [9, 10]. Such abnormal microenvironment is not beneficial for the recruitment and gravitation of immune cells to endometrial stromal and glandular areas [11, 12]. Consequently, persistent inflammation hampers endometrial receptivity and causes infertility [13, 14]. Hence, improving the endometrial immune microenvironment and promoting endometrium repair to enable implantation are the key requirements for CE therapy. There are various types of immune cells in the human endometrium. Macrophages, as an important type of immune cell, secrete pro-inflammatory and anti-inflammatory cytokines, which are crucial for maintaining menstruation and re-establishing the endometrial functional layer to create an appropriate microenvironment for embryo implantation [15, 16]. During pregnancy, macrophages are present in the maternal–fetal interface, and macrophage polarization is essential for embryo growth [17]. Abnormal macrophage regulation has been observed in CE patients and may be a factor leading to reproductive failure [18]. Therefore, it is necessary to find a safe and effective method to cure CE through macrophage modulation and improve the endometrial microenvironment for fertility.

Collagen, one of the major components of the extracellular matrix (ECM), not only maintains the structure of tissues but also participates in physiological processes. To date, 29 types of collagen have been identified in humans, including fibrous collagen (I, II, III, V and XI) and non-fibrous collagen, and they are mainly distributed in skin, ligaments and tendons [19]. Collagen is also an important component of the endometrium, and it plays a crucial role in successful pregnancy. In the endometrium of human and rat, type I, III and V collagen distributed predominantly. After embryo implantation, type III collagen is an abundant collagen component in the maternal–fetal interface of rat which might be associated with trophoblast attachment and invasion [20–25]. Abnormal expression or metabolism of collagen might cause tissue dysfunction and immune imbalance, leading to pathological pregnancy [26, 27]. Since the 1880s, researchers have investigated the application prospects of collagen [28]. As a starting material of medical devices, native collagen obtained from appropriate technology has good biocompatibility and low cytotoxicity, and it is capable of performing different influences on cell functions, like cell adhesion, cell migration and signal transduction [29]. Thus, collagen has become an ideal biomaterial widely applied in wound healing, cardiovascular system, tissue regeneration and drug delivery systems [30–33]. In addition, collagen-based biomaterials have been demonstrated to induce an anti-inflammatory macrophage response and promote tissue regeneration [34–37]. However, animal-derived collagen has risks of virus transmission, potential immunogenicity and possibility of inflammation caused by degradation [38]. Thus, recombinant humanized collagen developed by biosynthesis technology and featured by tandem repeat of amino acid unit, which was encoded by a specific segment of human collagen gene [39], was an attractive alternative and competitive strategy to animal-derived collagen. As type III collagen could combine with plentiful receptors expressed by immune cells and regulate immune cells activities [40–43], a recombinant humanized collagen type III (rhCol III) was used in this study. The amino acid sequence of the repeat unit in rhCol III was exactly same as that in human type III collagen (Gly483–Pro512). In previous studies, rhCol III showed favorable characteristics and material-cell interactions, including high cell adhesion ability, good solubility and great biocompatibility [44, 45]. Also, it had shown the capability to promote the regeneration of atrophic vaginal epithelium, improve pelvic floor function and repair damaged UV-photoaging skin [46–48].

Considering the crucial role of macrophages in CE and the immune regulation ability of collagen, rhCol III might be an effective method for CE treatment. In this study, macrophage-conditioned medium and lipopolysaccharide (LPS) induced inflammatory endometrium stromal cells (ESCs) model was firstly established. To study the regulation of rhCol III on macrophage polarization and the secretion of pro-inflammatory and anti-inflammatory cytokines by ESCs, the cell behaviors, decidualization and cell metabolism were observed, and the interactions between collagen and discoidin domain receptors (DDRs) were explored. Furthermore, the possible involved molecular mechanism of the NF-κB/YAP signaling pathway was further discussed. An LPS-induced endometritis model was established for an *in vivo* study, and rhCol III was applied for endometrial infusion. Histological manifestation, inflammatory response, macrophage modification and ECM remodeling were utilized to evaluate the effect of rhCol III on endometrial reconstitution.

## Materials and methods

### Preparation of rhCol III

Recombinant humanized collagen type III (rhCol III) was prepared as previously described [44]. Briefly, the amino acid sequence of rhCol III was designed from native human type III collagen (hCOL3A1, Gly483-Pro512) and composed of 16 tandem repeats of the 30 amino acid triple-helix fragments. The sequence was already verified and recorded with accession number of 6A0A and 6A0C in Protein Data Bank (PDB). The *Escherichia coli* (BL21 (DE3)) containing the rhCol III gene was induced with isopropylthio-β-galactoside and purified by chromatography to obtain endotoxin-free rhCol III. The lyophilized rhCol III was dissolved in sterilized phosphate-buffered saline (PBS) before use. The scanning electron microscopy (SEM; Hitachi, Japan) was used to observe the microstructure and morphology of rhCol III. For SEM examination, rhCol III fibers were sputtered with a layer of gold.

### Cell experiments

#### Cell extraction

The methodology for collecting endometrium samples from patients was reviewed and approved by the Ethics Committee of The Second Affiliated Hospital of Chongqing Medical University. Ten patients with mean age 30 ± 6.2 years who underwent hysteroscopy because of abnormal uterine bleeding were enrolled. Endometrium was obtained by mechanical scraping and stored in 40 ml 1× Hank's balanced salt solution (HBSS) with 100 U/ml penicillin and 100 µg/ml streptomycin. Endometrium was washed with fresh HBSS for 3–5 times to remove blood and mucous. The tissues were cut into 1 × 1 cm^2^ samples with sterilized scissors. The samples were collected and digested with 20 ml RPMI-1640 (Gibco, USA) medium containing type I collagenase (2.5 mg/ml; Sigma, USA) and DNase I (0.5 mg/ml; Sigma) for 1 h at 37°C. The mixture containing the digested samples was filtered through a 40-µm cell strainer. The resulting cell suspension was centrifuged at 800×*g* for 3 min and the supernatant was gently removed, and then, this process was repeated for a second time. Next, the cells were resuspended in complete RPMI-1640 medium supplemented with 10% fetal bovine serum (FBS; Gibco) and 1% penicillin (10 000 U/ml) and streptomycin (10 000 µg/ml). The cells were plated in 100-mm dishes and incubated in a 5% CO_2_ incubator at 37°C. The medium was changed every 2 days.

#### Cell culture

The THP-1 cell line was used for macrophage differentiation and obtained from the Cell Bank of the Chinese Academy of Sciences (Shanghai, China). THP-1 cells were cultured in complete RPMI-1640 medium (10% FBS and 1% penicillin/streptomycin). For M0 differentiation, THP-1 cells were treated with 100 nM phorbol 12-myristate 13-acetate (PMA; Sigma) for 48 h. Then, M0 macrophages were incubated with IFN-γ (20 ng/ml; Petrotech, UK) and LPS (100 ng/ml) for 48 h, which could induce macrophages to M1 phenotype. At the same time, rhCol III (0, 10, 100 and 1000 μg/ml) was added. The medium and cells were collected for subsequent steps.

ESCs were induced by 100 μg/ml LPS for 48 h to establish a cellular inflammatory model. To mimic the inflammatory response *in vivo*, the M1 macrophages cultured medium was added to co-culture with LPS stimulation ESCs for 48 h. Six groups set during the *in vivo* ESCs culture course, namely the NC group, NC + 100LPS group (100 μg/ml LPS stimulation), NC + 100LPS + M1 group (100 μg/ml LPS stimulation and M1 macrophages cultured medium), 10-rhCol III group, 100-rhCol III group and 1000-rhCol III group (in the latter three groups, 10, 100 and 1000 μg/ml rhCol III was added in the LPS stimulation and M1 macrophages cultured medium, respectively).

#### Cell adhesion

rhCol III was diluted in PBS at final concentration of 1000 μg/ml. Collagen solution (100 µl per well) was added and coated on 96-well culture plates at 37°C for 2 h in an incubator. After that, the solution was removed slightly and blocked by 5% bovine serum albumin (BSA) at 37°C for 1 h. ESCs (5 × 10^4^ cells/well) were plated and incubated at 37°C for 1 h and washed with PBS for three times. Cell counting kit-8 (CCK-8; MCE, USA) was used to measure cell adhesion rate on a microplate reader (Thermo Scientific, USA).

#### Cell proliferation

ESCs were plated in a 96-well cell culture plate (4 × 10^4^ cells/well). After incubation for 48 h, the proliferation of different groups was evaluated by CCK-8 and measured with a microplate reader (Thermo Scientific).

#### Cell migration

Briefly, ESCs (2 × 10^5^ cells/well) were seeded in a six-well plate for 48 h and reached a confluency of almost 90%. Scratches were made with a 10-μl pipette tip, and then, the plate was washed with PBS three times to remove scratched cells. RPMI-1640 medium was added to each well. At two time points (0 and 48 h), cells in the scratched area were recorded using an inverted microscope (Nikon, Japan).

#### Flow cytometry

According to the manufacturer’s instructions, macrophage polarization was detected by flow cytometry. Briefly, at least 1 × 10^7^ macrophages were detached and incubated with antibodies. Primary antibodies against cluster of differentiation 80 (CD80) (M1 macrophage marker, BioLegend, US), CD86 (M1 macrophage marker, BioLegend, US), CD163 (M2 macrophage marker BioLegend, US) and CD206 (M2 macrophage marker BioLegend, US) were used to identify the macrophage polarization. After incubation at 4°C for 1 h, cells were washed three times with PBS and evaluated with a flow cytometer (CytoFLEX; Beckman Coulter, USA). The data were analyzed using FlowJo software (TreeStar, USA).

#### In vitro decidualization assay

For decidualization, different groups of ESCs (1 × 10^6^ cells/well) were cultured with phenol red-free DMEM/F12 containing 2% charcoal stripped fetal bovine serum (Gibco), 10 nM estradiol (E2; Sigma), 1 µM medroxyprogesterone acetate (MPA; Sigma) and 50 µM cyclic adenosine monophosphate (Sigma) in six-well plates. Cells were incubated in a 5% CO_2_ incubator at 37°C for 48 h. Decidualization was evaluated by the expression of prolactin (PRL) and insulin-like growth factor binding protein-1 (IGFBP-1) [49] *via* quantitative polymerase chain reaction (q-PCR) analysis.

#### Western blotting

ESCs and macrophages were extracted by precooled RIPA lysis buffer containing 1% protease and phosphatase inhibitors (Beyotime, China). Total proteins of cell concentration were calculated using a BCA Protein Assay Kit (Beyotime). Proteins were separated by SDS-PAGE (8–12%) and transferred onto 0.45 μm PVDF membranes (Merck Millipore, USA). Then, the membranes were blocked with 5% (w/v) BSA solution for 2 h at room temperature. Next, the membranes were incubated with primary antibodies (Supplementary Table S1) at 4°C overnight. The membranes were washed with TBST solution three times and then incubated with secondary antibodies (1:10 000, Proteintech, China) for 2 h at room temperature. The signals of proteins were visualized by enhanced chemiluminescence kit (ECL, CA) and exposed with a ChemiDoc XRS system (Bio-Rad, USA). The protein expressions were normalized by GAPDH, and images were analyzed by Image Lab software (Bio-Rad).

#### Metabolite extraction and analysis

ESCs (1 × 10^7^/flask) were cultured in a 75-cm^2^ flask that contained M1 cultured complete medium and 100 μg/ml LPS for 48 h. In the rhCol III treatment groups, rhCol III was added to reach the final concentrations of 10, 100 and 1000 μg/ml. Then, the flask was washed with PBS twice and 1 ml prechilled 80% methanol was added to resuspended cells. The samples were chilled on ice for 30 s and centrifuged at 5000 rpm, 4°C for 1 min. Next, the cell supernatant was freeze-dried and dissolved with 10% methanol. Finally, the solution was analyzed using the UHPLC–MS/MS system (Thermo Fisher, Germany). For UHPLC–MS/MS analysis, samples were injected onto a Hypesil Gold column (100 mm × 2.1 mm, 1.9 μm) with a 12-min linear gradient at a flow rate of 0.2 ml/min. For the positive polarity mode, eluent A was 0.1% FA in water and eluent B was methanol. The data files were processed using the Compound Discoverer 3.1 (CD3.1; Thermo Fisher) to perform peak alignment, peak picking and quantitation for each metabolite. Principal component analyses (PCA) were performed at metaX. These metabolites were annotated using the KEGG and HMDB databases [50, 51]. Volcano plots were applied to filter metabolites based on log_2_ (FoldChange) and −log_10_ (*P*-values) by ggplot2 in R language of metabolites. The data of clustering heat maps were normalized by *z*-scores and plotted by Pheatmap package in R language. The results were analyzed using the statistical software R (version-3.4.3), Python (2.7.6 version) and CentOS (6.6 version).

### Animal experiments

#### Animals

All animal experiments were handled humanely and were approved by the Ethics Committee of Chongqing Medical University. Four- to six-week-old female (120–180 g) and 9- to 11-week-old male (300–400 g) Sprague-Dawley (SD) rats were purchased from the Experimental Animal Center of Chongqing Medical University. All rats were fed in plastic cages under the temperature range of 20–26°C, with 50–60% humidity and 12 h light/dark cycles.

#### Animal models

Cycle synchronized analysis was conducted by vaginal smear for female SD rats after allowing about 2 weeks for acclimatization. Rats at the estrus period were used for the *in vivo* study. To establish the CE model, LPS (*E. coli* 055: B5; Sigma) was dissolved in sterilized PBS solution at 5 mg/ml concentration [52]. Briefly, the uteri were exposed after anesthesia, and 100 μl LPS solution was infused into each rat’s uteri. One hundred and twenty rats were randomly divided into four groups: negative control group (NC group), model group, antibiotic group and rhCol III group. In the antibiotic, rhCol III and model groups, 24 h after LPS solution infusion, 100 μl levofloxacin solution (10 mg/ml), 100 μl rhCol III solution (10 mg/ml) or 100 μl sterilized PBS solution was infused in each rat’s uteri. In the NC group, each rat’s uterus was infused with 100 μl sterilized PBS solution two times at 24-h intervals. Rats were sacrificed on Days 1, 4, 7, 14 and 28 after the second infusion, and both uteri were harvested from each rat.

#### FITC-labeled rhCol III and tracking of collagen transplantation

Recombinant humanized type III collagen (rhCol III, 4 mg) and fluorescein isothiocyanate (FITC, 2 mg; Bioss, China) were dissolved and stirred in saturated sodium bicarbonate solution (pH 8–9) under a dark environment at 4°C overnight. Then, the solution was dialyzed at 4°C for 48 h. After FITC-labeled rhCol III was infused into the uterus, at the time points of 2 h and Days 1, 2, 4, 7, 14, 28 and 60, uterine tissues were obtained and fixed with optimal cutting temperature compound (OCT; SAKURA, Japan) at 4°C and then cut into 10-μm slices. The sections were observed under a fluorescence microscope at a 100× magnification (Nikon Corporation, Tokyo, Japan).

#### Histological analysis

Uterine tissues were collected and fixed in 4% paraformaldehyde, embedded in paraffin and cut into 5-μm slices. According to the manufacturer’s instructions, sections were stained with hematoxylin and eosin (H&E; Solarbio, China). Four high-power fields (HPFs) of each section were randomly selected to measure endometrial thickness under a 100× magnification of an optical microscope (Nikon Corporation, Tokyo, Japan). Uterine endometrial thickness, that is, the vertical distance from the serous membranes to the luminal surface, was measured by ImageJ software. The numbers of glands, neutrophils and macrophages were counted and recorded for each HPF.

#### Immunohistochemistry

Briefly, the paraffin sections were dried at 60°C for 2 h and deparaffinized by xylene for 40 min. Then, the sections were dehydrated with gradient alcohols and subjected to antigen retrieval. Next, the sections were washed with PBS three times, treated with endogenous peroxidase blocker (SPlink Detection Kits, ZSGB-BIO) and incubated at 37°C for 10 min. Subsequently, the sections were washed with PBS three times and blocked with goat serum for 20 min at 37°C. The sections were incubated with primary antibody, including anti CD138 (1:200; Bioss) and anti HOXA10 (1:200; Bioss), at 4°C overnight. After being rewarmed at 37°C for 30 min and washed with PBS three times, the sections were incubated with the secondary antibody at 37°C for 20 min. Then 3,3′-diaminobenzidine staining was added to enable visualization. Three random HPFs were selected from each section under 200× magnification, and the images were analyzed by ImageJ software.

#### Immunofluorescent staining

Paraffin sections were deparaffinized and dehydrated. Next, the sections were blocked with 10% goat serum for 1 h at 37°C after antigen retrieval. The sections were incubated with primary antibodies, including anti-F4/80 (1:200; Bioss), anti-CD86 (1:200; Bioss) and anti-CD163 (1:200; Santa Cruz, USA), overnight at 4°C. After being washed three times, the sections were incubated with fluorescent-conjugated secondary antibodies (1:200; CST, USA) for 1 h at 37°C and then 4′,6-diamidino-2-phenylindole solution was used for nuclear staining. The sections were observed under a fluorescence microscope at 400× magnification (Nikon Corporation, Tokyo, Japan).

#### Fertility test

For the fertility test, 120 female and 60 male rats were used. The groups were divided as previously described. Different groups of rats were mated with male rats (2:1) at 1, 4, 7, 14 and 28 days after infusion at night. Vaginal plug was confirmed for female rats in the following morning after mated with male rats and the day vaginal plug was observed was considered as embryonic stage E 0.5. The pregnant rats were sacrificed at E 13.5, and the number of embryos was counted.

### Quantitative real-time PCR

RNA from tissues and cells was extracted according to the manufacturer’s instructions (Takara, Japan), and total RNA was isolated with 1 ml TRIzol reagent (Invitrogen, USA). In reverse transcription reaction, 1 μg RNA was used for complementary DNA synthesis with PrimeScript RT Master Mix. The relative quantification of mRNA was performed with gene primers and TB Green Premix Ex Taq II using the CFX96 Real-Time system (Bio-Rad). Each reaction was performed three independent times. The expression of genes was normalized by using the GAPDH gene as the control. The relative quantification was valued using the formula 2^−ΔΔ^CT, and the sequence of primers is listed in Supplementary Table S2.

### Statistical analysis

All data were presented as the mean ± standard deviation (SD), and at least triplicate tests were performed. One-way analysis of variance was used for multiple comparisons, and Student’s *t*-test was applied for the comparison between two groups. Data were analyzed using SPSS software (version 22.0, IBM, Armonk, NY, USA). *P* < 0.05 was defined as indicating a significant difference.

## Results

### rhCol III regulated macrophage polarization and inflammatory cytokines *in vitro*

As described in literatures [44, 45], rhCol III is composed of 16 tandem repeats of the hCOL3A1 483–512 segment (Supplementary Fig. S1A) and this segment shows high homology in rhCol III and animal collagen type III that could reduce immunogenicity when applied in animal model to observe its function (Supplementary Fig. S1B). Meanwhile, the cell adhesion ability of ESCs was promoted by rhCol III (Supplementary Fig. S1C), and the expressions of collagen type I and III were enhanced after rhCol III treatment (Supplementary Fig. S1D). From the result of SDS-PAGE, rhCol III was nearly 50 kDa and had a purity level (Supplementary Fig. S1E). The SEM figures of collagen fibers are presented in Supplementary Fig. S1F.

**Figure 1.**
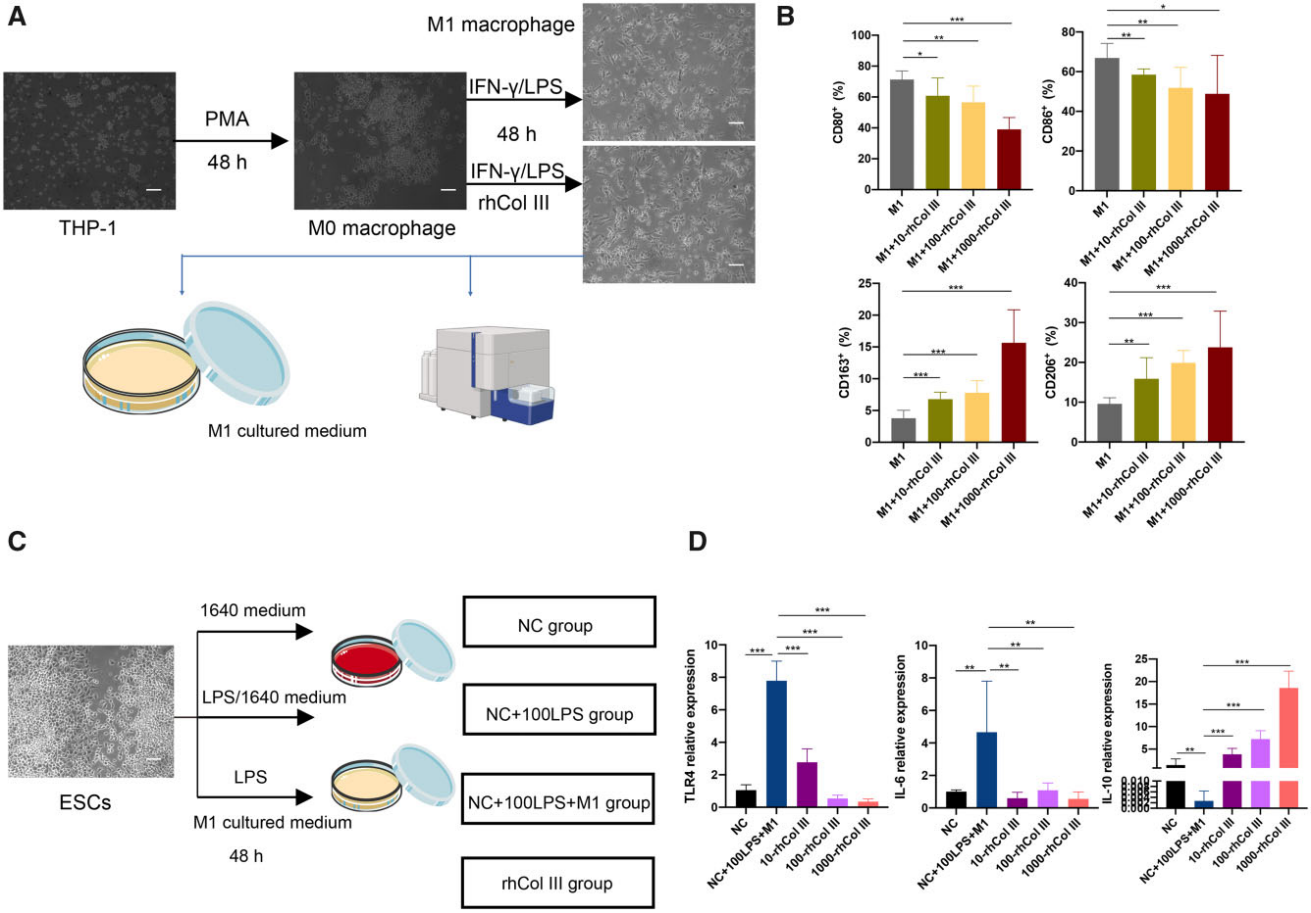
Immunomodulation effects of rhCol III *in vitro*. (**A**) THP-1 cells were directly differentiated with PMA to M0 and polarized toward M1 (IFNγ/LPS). (**B**) Statistical analysis of M1 macrophage marker (CD80, CD86) and M2 macrophage marker (CD206, CD163) by flow cytometry. (**C**) Schematic diagram of ESCs culture. (**D**) The expressions of pro-inflammatory genes (TLR-4, IL-6) and anti-inflammatory genes (IL-10) of ESCs in the NC, NC + 100LPS + M1, 10-rhCol III, 100-rhCol III and 1000-rhCol III groups through q-PCR analysis (*P < 0.05, **P < 0.01, ***P < 0.001).

*In vitro*, the modification properties of rhCol III on macrophage polarization were studied using M1 macrophages stimulated by IFNγ/LPS from M0 macrophages differentiated from PMA-induced THP-1 cells (Fig. 1A).

The macrophage phenotypes, M1 and M2, were studied by flow cytometry for CD86, CD80 (marker for M1) and CD163, CD206 (marker for M2). In the M1 group, M1 macrophages secreted more CD86 and CD80 and less CD206 and CD163 (Supplementary Fig. S2A). In the M1 and rhCol III-cultured group, the expression of CD86 and CD80 decreased and the expression of CD206 and CD163 increased after rhCol III treatment compared with the M1 group. The extent of macrophage polarization toward M2 phenotype was in direct proportion to increased rhCol III concentration (Fig. 1B).

To mimic the inflammation microenvironment of CE, two methods were applied to establish cell models, the first being LPS-stimulated ESCs and the second being M1 macrophages cultured medium and LPS-stimulated ESCs (Fig. 1C). The expressions of pro-inflammatory cytokines [toll-like receptor 4 (TLR-4) and interleukin 6 (IL-6)] and anti-inflammatory cytokines (IL-10) of ESCs were examined by q-PCR. According to the results of TLR-4, IL-6 and IL-10 expression, LPS could induce the inflammation reaction since the expressions of TLR-4 and IL-6 increased and the expression of IL-10 decreased in the NC + 100LPS group compared with the NC group (Supplementary Fig. S2B). In addition, the M1 macrophages cultured medium and LPS coordinately upregulated the expressions of inflammation cytokines TLR-4 and IL-6 and downregulated the expression of IL-10 compared with only LPS, which might illustrate that co-culture of LPS and M1 macrophages cultured medium better simulates the pathological environment *in vivo*. After treatment with rhCol III, the expressions of TLR-4 and IL-6 were lower compared to the NC + 100LPS + M1 group. Conversely, the expression of IL-10 was lower in the NC + 100LPS + M1 group compared to the NC and rhCol III groups (Fig. 1D).

### rhCol III promoted cell migration, viability and decidualization *in vitro*

**Figure 2.**
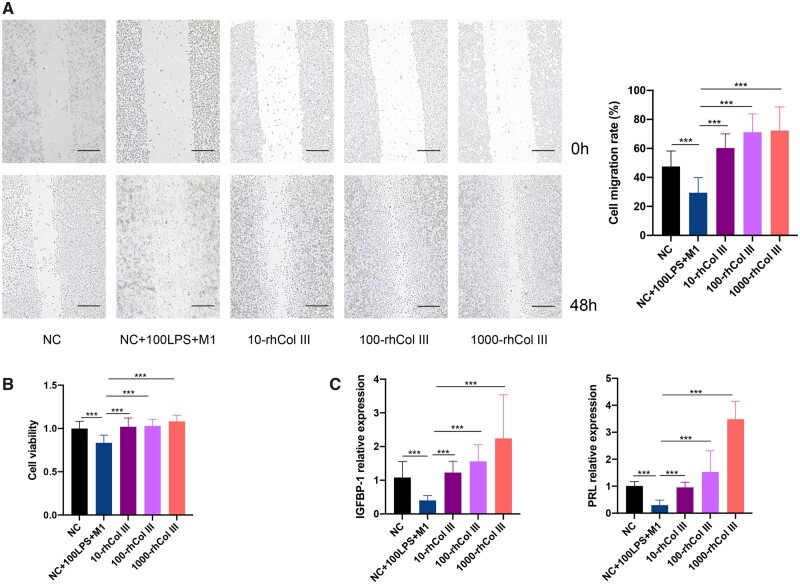
The biological functions of rhCol III on cell migration, viability and decidualization. (**A**) Representative images of cell scratch assay at 100× magnification of two time points (0 and 48 h) and statistical analysis of cell migration rates over the course of 48 h in the NC, NC + 100LPS + M1, 10-rhCol III, 100-rhCol III and 1000-rhCol III groups. Scale bar = 100 μm. (**B**) Cell viability in the NC, NC + 100LPS + M1, 10-rhCol III, 100-rhCol III and 1000-rhCol III groups after 48 h incubation. (**C**) The expression levels of IGFBP-1 and PRL by q-PCR of the NC, NC + 100LPS + M1, 10-rhCol III, 100-rhCol III and 1000-rhCol III groups (***P < 0.001).

To detect the function of rhCol III on cell biological behavior, the cell migration and viability of ESCs were evaluated. Figure 2A presents the migration ability of rhCol III treatment over the course of 48 h. Stimulated by LPS or LPS and M1 macrophages cultured medium, cell migration ability was suppressed compared with the NC group, and there was no significant difference between the NC + 100LPS and NC + 100LPS + M1 groups (Supplementary Fig. S3A). With the treatment of rhCol III at three different concentrations, the cell migration ability increased compared to the NC + 100LPS + M1 group (Fig. 2A). The result of cell viability was similar to the result of cell migration, as culture with LPS or LPS + M1 medium restrained the cell proliferation ability (Supplementary Fig. S3B). In addition, rhCol III eliminated the inhibition effect and promoted cell viability compared with the NC + 100LPS + M1 group (Fig. 2B).

IGFBP-1 and PRL were the two gene markers used to evaluate the decidualization process *in vitro*. Thus, the gene expressions of IGFBP-1 and PRL were detected by q-PCR to verify the effect of rhCol III on decidualization improvement. The expressions of IGFBP-1 and PRL decreased in both the NC + 100LPS group and NC + 100LPS + M1 group compared with the NC group, and there was no significant difference between these two groups (Supplementary Fig. S3C). However, in the rhCol III groups, the expressions of IGFBP-1 and PRL were upregulated compared to the NC + 100LPS + M1 group (Fig. 2C).

### rhCol III regulated collagen synthesis and DDR expression and suppressed activation of NF-κB/Yap signaling pathway

**Figure 3.**
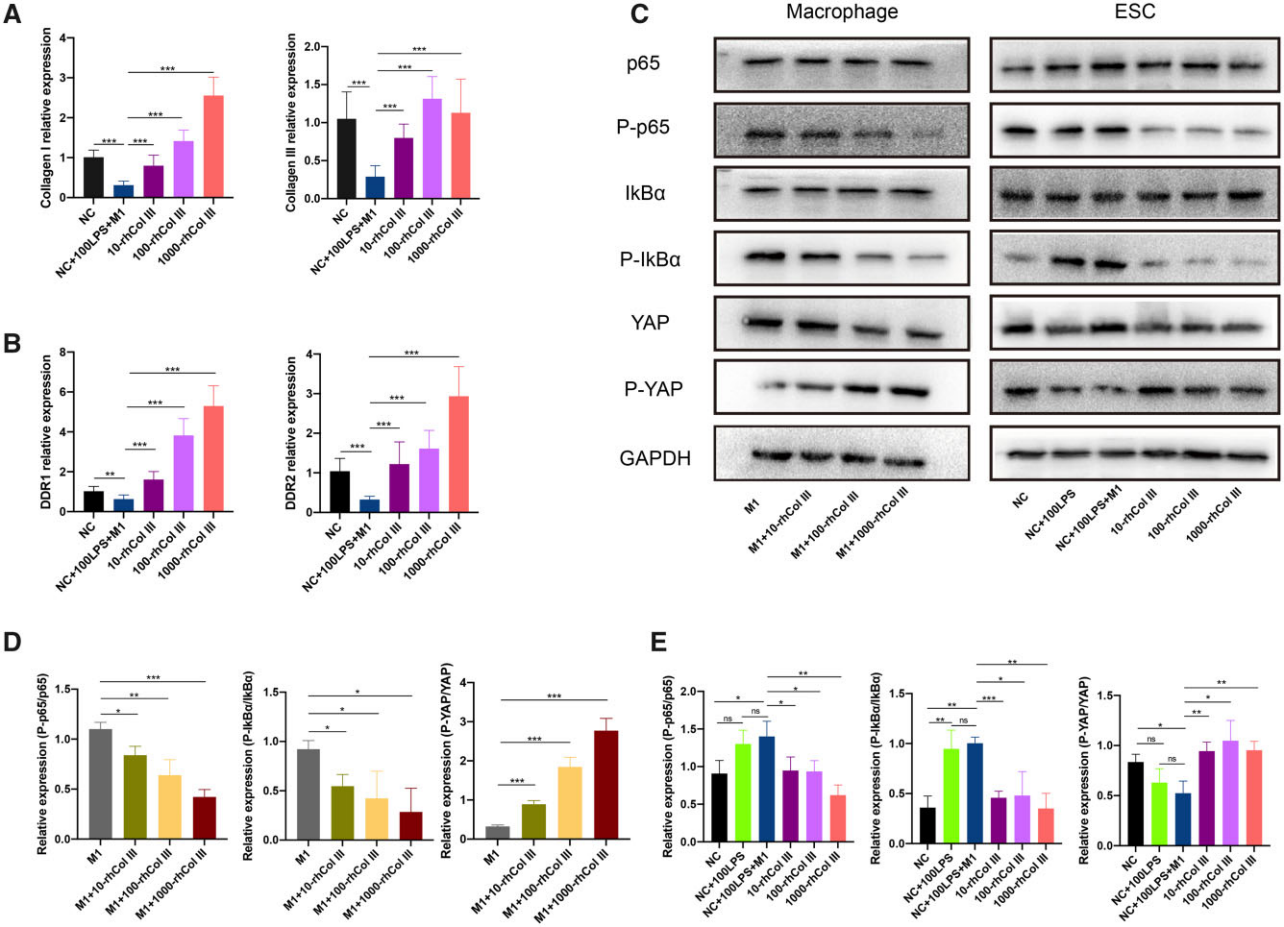
*In vitro* analysis of the effects of rhCol III in regulating the expression of collagen and DDR and the NF-κB/Yap signaling pathway. (**A**) The expression levels of collagen I and III determined by q-PCR of the NC, NC + 100LPS + M1, 10-rhCol III, 100-rhCol III and 1000-rhCol III groups. (**B**) The expression levels of DDR1 and DDR2 determined by q-PCR of the NC, NC + 100LPS + M1, 10-rhCol III, 100-rhCol III and 1000-rhCol III groups. (**C**) The protein expression of IκBα, P-IκBα, p65, P-p65, Yap and P-Yap in the M1, M1 + 10-rhCol III, M1 + 100-rhCol III, M1 + 1000-rhCol III groups and NC, NC + 100LPS, NC + 100LPS + M1, 10-rhCol III, 100-rhCol III and 1000-rhCol III groups detected by Western blotting. (**D**) The protein expression levels of IκBα, P-IκBα, p65, P-p65, Yap and P-Yap in the M1, M1 + 10-rhCol III, M1 + 100-rhCol III and M1 + 1000-rhCol III groups determined by Western blotting. (**E**) The protein expression levels of IκBα, P-IκBα, p65, P-p65, Yap and P-Yap in the NC, NC + 100LPS, NC + 100LPS + M1, 10-rhCol III, 100-rhCol III and 1000-rhCol III groups determined by Western blotting (*P < 0.05, **P < 0.01, ***P < 0.001).

To assess the effect of rhCol III on collagen synthesis, the expressions of collagen I and III by ESCs were examined *in vitro* by q-PCR. For collagen I level, the expression decreased in the NC + 100LPS and NC + 100LPS + M1 groups compared to the NC group, and the expressions of the former two groups were similar (Supplementary Fig. S3D). Regarding treatment with rhCol III, the gene expression increased gradually with the increase in concentration of rhCol III (Fig. 3A). Notably, only LPS stimulation promoted collagen III expression by ESCs, and the expression decreased after co-culture with M1 macrophages cultured medium (Supplementary InformationFig. S3D). Compared with the NC + 100LPS + M1 group, collagen III expression increased in the rhCol III groups (Fig. 3A). There was a slight decrease in the collagen III level in the 1000 μg/ml rhCol III group compared to the 100 μg/ml rhCol III group, and it approached the level of the NC group. DDRs are receptors activated by specific collagen types, and they play important roles in cell adhesion and migration and matrix remodeling. According to q-PCR analysis, the expressions of DDR1 and DDR2 were both downregulated in the NC + 100LPS and NC + 100LPS + M1 groups compared with the NC group, and the expression of DDR1 showed no statistical difference between the two former groups (Supplementary Fig. S3E). After treatment with rhCol III, the expressions of DDR1 and DDR2 were upregulated compared with the LPS + M1 medium stimulation group (Fig. 3B).

To further explore the mechanism involved in immune responses regulation, the effects of rhCol III on the NF-κB and YAP signaling pathways were investigated. The NF-κB signaling pathway is related to inflammation. The regulation activity of rhCol III on M1 macrophages through the NF-κB pathway was examined first (Fig. 3C). The protein expressions of phosphorylated p65 and IκBα were observed by western blotting. In the rhCol III groups, p-p65 and p-IκBα were downregulated, and the expressions decreased gradually with increasing rhCol III concentration. The expressions of these two proteins in the M1 group were higher than in the other three groups (Fig. 3D). Then, the expressions of these two phosphorylated proteins were observed in ESCs (Fig. 3E). As shown in Fig. 3E, the expressions of p-p65 and p-IκBα were increased in the NC + 100LPS and NC + 100LPS + M1 groups compared with the NC group and the three rhCol III groups. There were no significant differences in p65 or IκBα expressions between any groups.

YAP is a co-effector in the Hippo signaling pathway and contributes to the innate immune response. Therefore, the expressions of YAP and p-YAP by M1 macrophages and ESCs were observed (Fig. 3C). The inhibition of rhCol III on YAP protein was similar with p-p65 and p-IκBα; its expression was increased in the M1 group compared with the rhCol III groups (Fig. 3D). In the NC + 100LPS and NC + 100LPS + M1 group, the expression of YAP was higher than NC and the other three rhCol III groups. On the contrary, p-YAP protein was downregulated in the NC + 100LPS and NC + 100LPS + M1 group compared with the NC and rhCol III treatment groups. This indicated that rhCol III promoted YAP phosphorylation to the inactive YAP signaling pathway (Fig. 3E).

### rhCol III modulates cell metabolism

**Figure 4.**
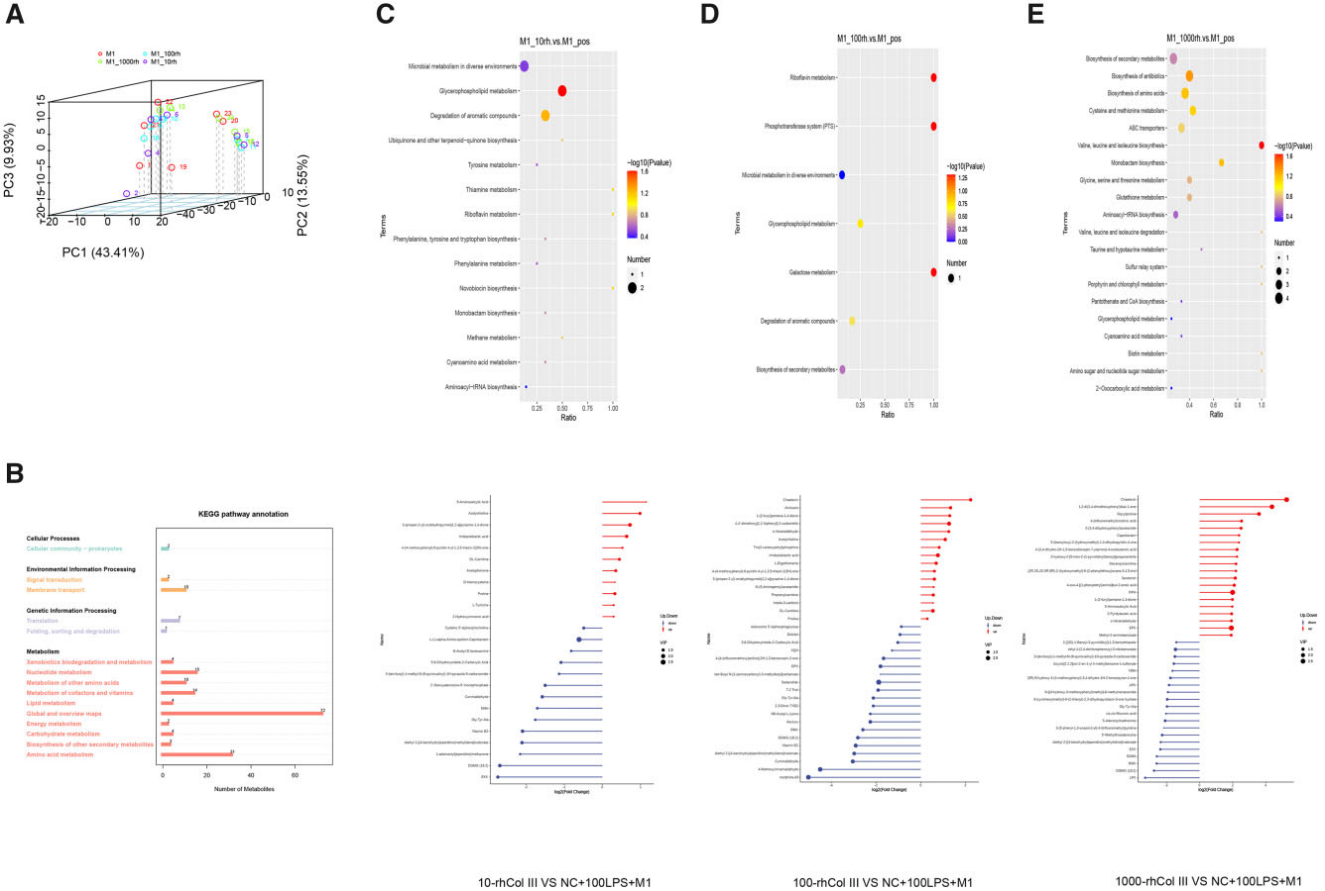
rhCol III modulated the metabolism of LPS and M1 macrophages medium stimulation ESCs after 48 h treatment. (**A**) PCA of the NC + 100LPS + M1, 10-rhCol III, 100-rhCol III and 1000-rhCol III groups. (**B**) KEGG pathway analysis of differential metabolites. (**C**) Differential metabolites in the 10-rhCol III groups compared with the NC + 100LPS + M1 group. (**D**) Differential metabolites in the 100-rhCol III groups compared with the NC + 100LPS + M1 group. (**E**) Differential metabolites in the 1000-rhCol III groups compared with the NC + 100LPS + M1 group.

After M1 macrophage medium co-cultured ESCs were treated with rhCol III for 48 h, cell metabolites were detected by the LC–MS/MS system. PCA demonstrated the distributions of metabolites in different groups (Fig. 4A). A total of 440 metabolites were identified. In total, 11, 16 and 46 metabolites were upregulated and 14, 23 and 53 metabolites were downregulated in the 10-rhCol III, 100-rhCol III and 1000-rhCol III groups compared with the NC + 100LPS + M1 group (Supplementary Fig. S4A). Compared with the NC + 100LPS + M1 group, the expression of proline or its derivative glycylproline increased in all three rhCol III treatment groups (Fig. 4C–E). Meanwhile, different metabolites showed upward trends, including dl-carnitine, acetylcholine and imidazolelactic acid in the 10-rhCol III (Fig. 4C) and 100-rhCol III (Fig. 4D) groups, 5-aminosalicylic acid in the 10-rhCol III and 1000-rhCol III (Fig. 4E) groups and chaetocin and o-veratraldehyde in the 100-rhCol III and 1000-rhCol III groups. The biosynthesis levels of amikacin and EPK were higher in the 100-rhCol III and 1000-rhCol III groups than in the NC + 100LPS + M1 group. The relationships among metabolites are shown in Supplementary Fig. S3B and C. In organisms, the coordinates of different metabolites are related with biological behaviors. Analysis of the KEGG pathway was performed to reveal biological functions (Fig. 4B). The results showed metabolites mainly participated in metabolism and environmental information processing. In the 10-rhCol III group, microbial metabolism in diverse environments and glycerophospholipid metabolism and degradation of aromatic compounds were upregulated compared with the NC + 100LPS + M1 group. In addition, riboflavin metabolism, phosphotransferase system and microbial metabolism in diverse environments were upregulated in the 100-rhCol III group *versus* the NC + 100LPS + M1 group. In the 1000-rhCol III group, biosynthesis levels of secondary metabolites, antibiotics and amino acids showed upward trends compared with the NC + 100LPS + M1 group.

### Effects of rhCol III on the histopathological changes of CE rats

**Figure 5.**
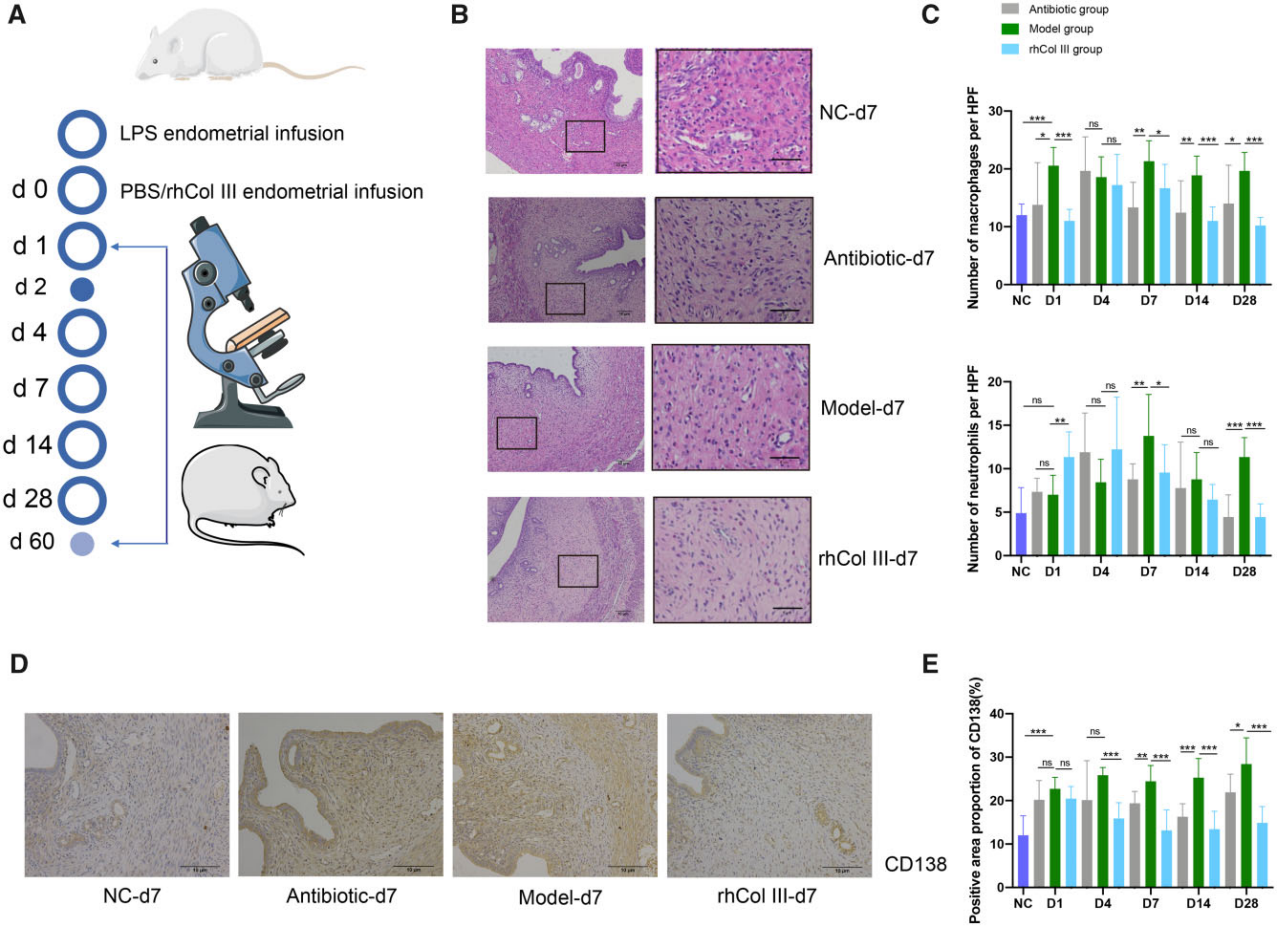
rhCol III suppressed the expression of CD138 and improved the histological features of CE rats. (**A**) Schematic illustration of animal experiments. (**B**) Endometrium samples were analyzed with H&E staining at 100× and 400× magnifications on Day 7 (scale bar = 10 or 5 μm). (**C**) Statistical analysis of macrophage and neutrophil numbers. (**D**) Immunohistochemical staining of CD138 in endometrium on Day 7 at 200× magnification (scale bar = 10 μm). (**E**) Statistical analysis of the area proportions of CD138 of NC, antibiotic, model and rhCol III groups (*P < 0.05, **P < 0.01, ***P < 0.001).

To establish the CE model, LPS solution was infused into each rat’s uterus for 24 h (Fig. 5A). The detection of CD138-positive cells in the endometrium is an effective method to diagnose endometritis. According to the results of immunohistochemical staining, CD138 expression was higher in the model group than in the NC group on Day 1, which proved LPS could induce the CE model (Fig. 5D and E). There was no significant difference in CD138 expression between the LPS, antibiotic and rhCol III groups on Day 1 (Supplementary Fig. S5A). From Days 4 to 28, CD138 expression in the endometrium of the rhCol III group showed a slight downward trend and was less than that in the model group at the same time point (Fig. 5E).

Histopathological changes were observed by H&E staining, the results of Day 7 were presented as representative in Fig. 5B and the remained H&E staining pictures are shown in Supplementary Fig. S5B. As shown in Fig. 5C, in the LPS group, inflammatory cells including neutrophils, macrophages and lymphocytes were diffused in the endometrium (Supplementary Fig. S5B). At the same time, endometrial congestion and edema appeared in the model group, which led to the endometrium becoming thicker than in the NC group. In addition, the gland number decreased compared with the NC group (Supplementary Fig. S5C). After rhCol III treatment, the endometrium became thinner and the gland number increased compared to the model group from Days 4 to 28 (Supplementary Fig. S5C). Only on Day 14, the endometrium was thinner in the antibiotic group than in the model group; on the other time point, two groups showed no significant difference. After rhCol III and antibiotic treatment, there were fewer macrophages than in the model group, except on Day 4 when two groups had a similar number of macrophages compared to the model group. More neutrophils were recruited in the model group during the 28-day observation. On Day 1, there were more neutrophils in the rhCol III group than in the LPS group (Fig. 5C). In the rhCol III and antibiotic groups, less neutrophils were present in the endometrium on Days 7 and 28 compared with the model group. The neutrophil numbers were similar at Days 4 and 14 among three groups (Fig. 5C).

### rhCol III immunomodulated macrophage polarization *in vivo* and reduced inflammation

**Figure 6.**
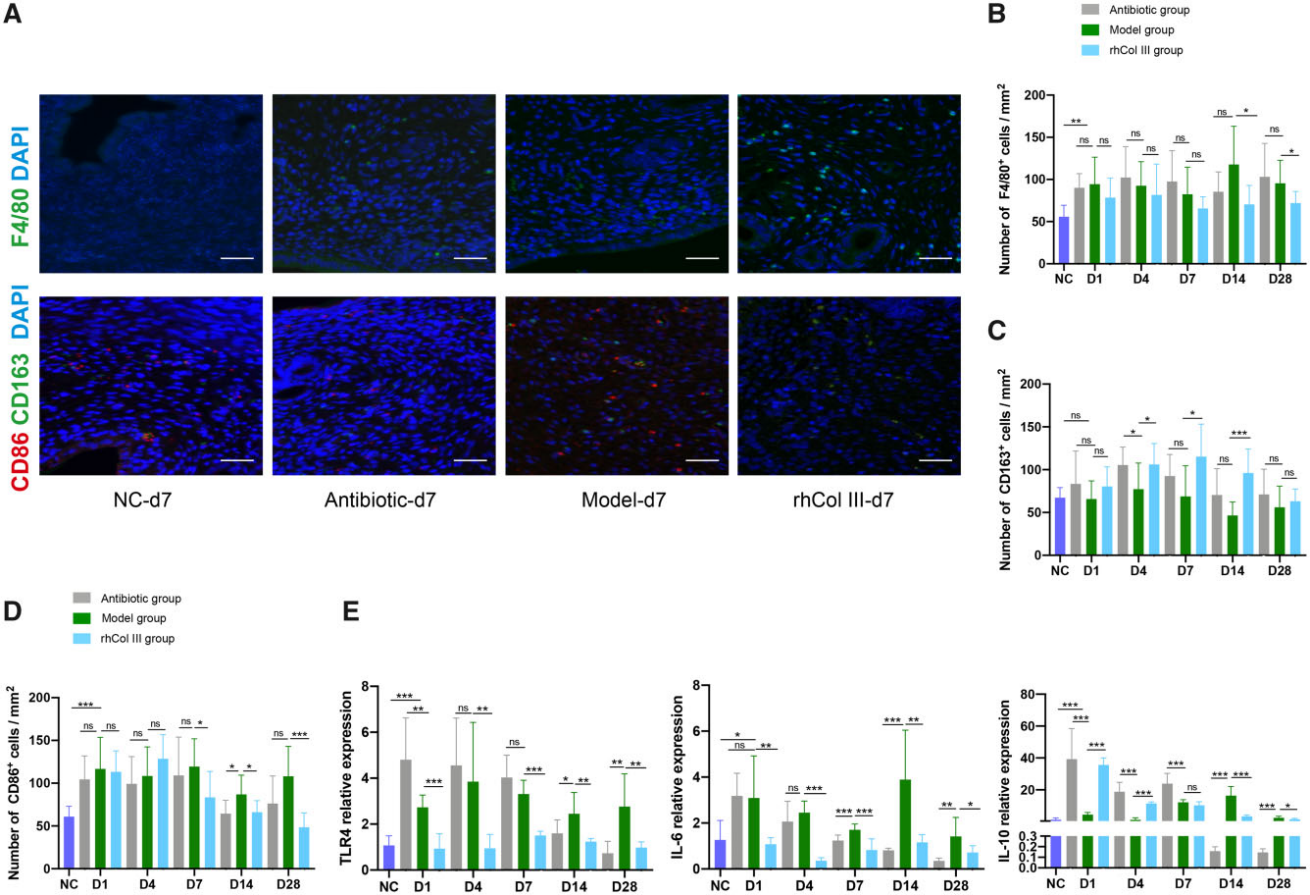
Effects of rhCol III on macrophage regulation and cytokines *in vitro*. (**A**) Immunofluorescence staining of endometrium for F4/80, CD86 and CD163 in four groups on Day 7 at 200× magnification. Scale bar = 100 μm. The staining color of F4/80^+^ cells was green. The staining color of CD86^+^ cells was red and that of CD163^+^ cells was green. (**B**) Quantification of F4/80^+^ cells in endometrium. (**C**) Quantification of CD163^+^ cells in endometrium. (**D**) Quantification of CD86^+^ cells in endometrium. (**E**) The relative expression of TLR-4, IL-6 and IL-10 of endometrium (*P < 0.05, **P < 0.01, ***P < 0.001).

Based on the results of H&E staining, the immune modulation mechanism of rhCol III might be related to the regulation of inflammatory cells. Macrophages play an important role in immunomodulation and tissue regeneration. The two phenotypes of macrophages (M1 and M2) have different biological functions. M1 produces pro-inflammatory cytokines, whereas M2 produces anti-inflammatory cytokines that exert an immunosuppressive effect. CD86 is the marker of the M1 phenotype, CD163 is the marker of the M2 phenotype, and F4/80 is the marker of macrophages, all of which were detected by immunofluorescence staining. The pictures of Day 7 in four groups were demonstrated in Fig. 6A and the photos of remained time points are seen in Supplementary Fig. S6A.

On Days 1–7, the number of F4/80-positive cells in the rhCol III group was similar to that in the model group. However, the number of F4/80^+^ cells in the model group was higher than that in the rhCol III group on Days 14 and 28 (Fig. 6B). Immunostaining for CD163 and CD86 is shown in Fig. 6C and D. More CD163^+^ cells were observed in the rhCol III group compared with the model group on Days 4, 7 and 14. There was no significant difference between the rhCol III group and the model group on Days 1 and 28, nor between the model group and the NC group on Day 1 (Fig. 6C). Regarding CD86^+^ cells, the number in the model group was higher than that in the NC group on Day 1. There was no significant difference between the model group and the rhCol III group on Days 1 and 4. On Days 7, 14 and 28, higher numbers of CD86^+^ cells were seen in the model group than in the rhCol III group (Fig. 6D). Compared with the model group, the numbers of all three types of cells were similar in antibiotic group except for the number of CD163-positive cells on Day 4 and the number of CD86 positive cells on Day 14.

At the same time, the endometrium of CE was diffused with cytokines. IL-6 and TLR-4 are pro-inflammatory cytokines, and IL-10 is an anti-inflammatory cytokine. In the rhCol III group, the expressions of TLR-4 and IL-6 were lower than in the model group during 28-day observation (Fig. 6E). However, the expression of IL-10 was increased in the rhCol III group compared with the model group on Days 1 and 4. IL-10 expression showed no significant difference between the model group and the rhCol III group on Day 7 (Fig. 6E). Interestingly, the expression of IL-10 in the model group increased on Day 14 and decreased on Day 28 but remained higher compared with rhCol III group. The expression levels of all three types of cytokines were different in the antibiotic group. On Day 1, the TLR-4 expression was higher in the antibiotic group than in the model group, and then, it decreased, nearing the level of the model group on Days 4 and 7. Later, it continued to decrease and was less than in the model group on Days 14 and 28. The expression of IL-6 was similar in the antibiotic group and the model group on Days 1 and 4. In the subsequent days, the expression of IL-6 decreased like in the rhCol III group. Regarding IL-10, its expression in the antibiotic group was higher than in the model group throughout Days 1–7. At Days 14 and 28, the expression of IL-10 was decreased in the antibiotic group than in the model group (Fig. 6E).

### rhCol III was localized in the endometrium and remodeled the ECM

**Figure 7.**
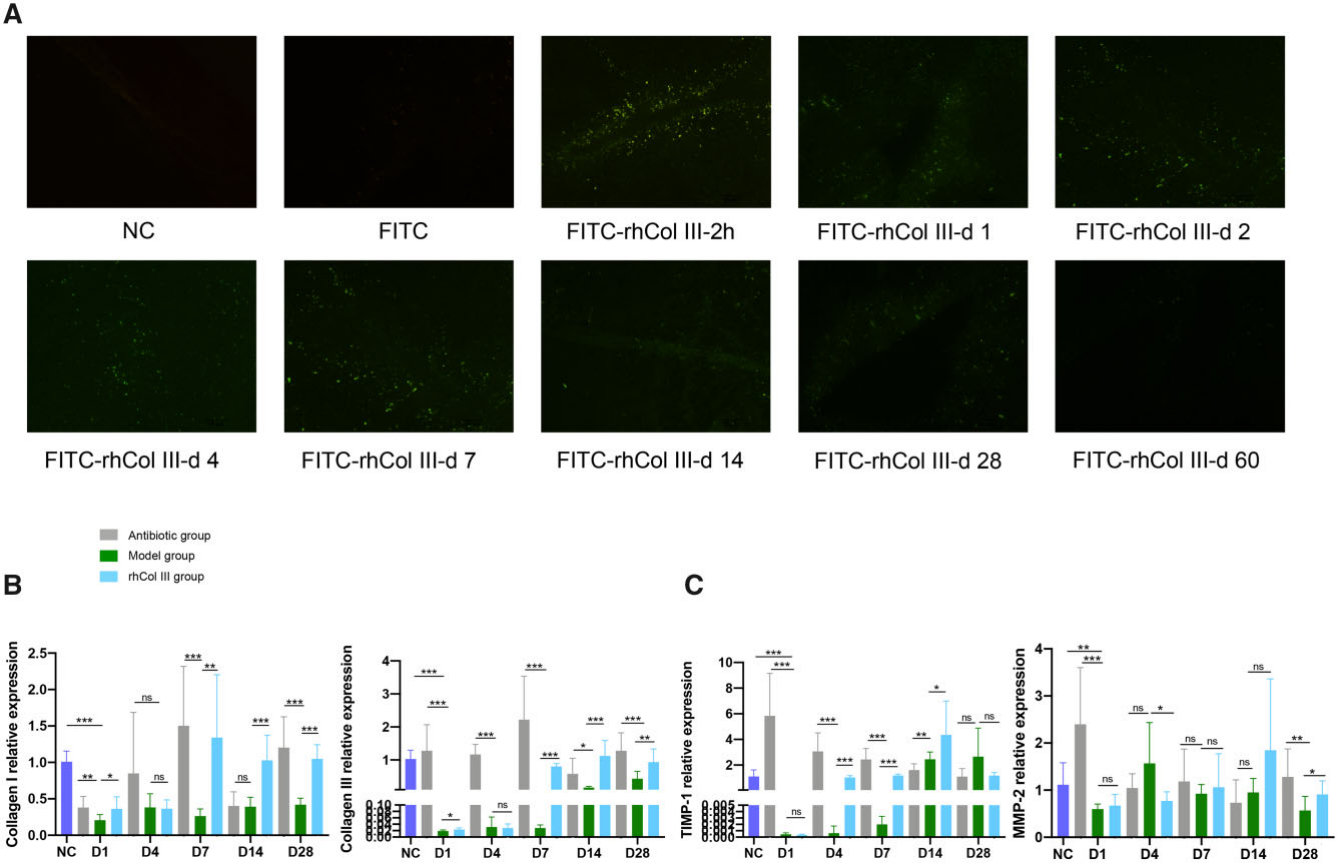
The tracking of rhCol III *in vivo* and evidence of rhCol III promoting ECM remodeling. (**A**) *In vivo* tracking of the location of FITC-rhCol III (green) at 100× magnification of different time points (2 h and Days 1, 2, 4, 7, 14, 28 and 60). Scale bar = 100 μm. (**B**) Statistical analysis of collagen I and III expression. (**C**) Statistical analysis of TIMP-1 and MMP-2 expression (*P < 0.05, **P < 0.01, ***P < 0.001).

To trace the localization of rhCol III *in vivo*, the FITC-labeled rhCol III was detected by immunofluorescence microscopy (Fig. 7A). At 2 h after treatment, the fluorescence signal was diffused around the epithelium layer of endometrium. From 24 h to Day 7 post-infusion, FITC-rhCol III could be seen throughout the whole endometrium. After Day 14, the FITC-rhCol III began to decrease, and little fluorescence was observed. On Day 60, the FITC-rhCol III was hardly found in the endometrium.

The ECM affords a dynamic structure for cells with tissues and is vital for regulating cell function and modulating immune responses. Collagen I and III are the main components of the ECM, and their expression levels reflect the ECM remodeling process. On Day 1, the expressions of collagen I and III were downregulated significantly in the model and rhCol III groups compared with the NC group. In the next time points, the expressions of collagen I and III showed upward trends, although the expressions were constantly higher in the rhCol III group than in the model group except on Day 4 (Fig. 7B). In the antibiotic group, the expression of collagen I was increased on Days 1, 7 and 28 compared with the model group. There was no significant difference between the antibiotic group and the model group at Days 4 and 14. Interestingly, the expression of collagen III in the antibiotic group was constantly higher than in the model group through whole process (Fig. 7B).

TIMP-1 and MMP-2 are two proteins involved in the remodeling of the ECM. As shown in Fig. 7C, TIMP-1 expression was reduced in the rhCol III and model groups compared with the NC group at Day 1. Then, the expression of TIMP-1 increased sharply in the rhCol III group compared with model group on Days 4, 7 and 14, followed by a decrease on Day 28 showed no significant difference with model group. The expression of TIMP-1 of the antibiotic group increased from Days 1 to 7 compared to the model group, whereas it decreased on Day 14. The expression of TIMP-1 in the antibiotic group was similar compared to the model group on Day 28, showing no significant difference. The MMP-2 expression was higher in the NC group than in the model group on Day 1. On Days 1, 7 and 14, there were no significant differences in MMP-2 expression between the rhCol III and model groups. The MMP-2 expression was increased in the model group on Day 4 compared to the rhCol III group. By contrast, the rhCol III group exhibited higher MMP-2 expression on Day 28 compared with the model group (Fig. 7C). On Days 1 and 28, the expression of MMP-2 was higher in the antibiotic group than in the model group, whereas at the other time points, there was no significant difference in MMP-2 expression between these two groups (Fig. 7C).

### rhCol III improved endometrium receptivity and restored fertility

**Figure 8.**
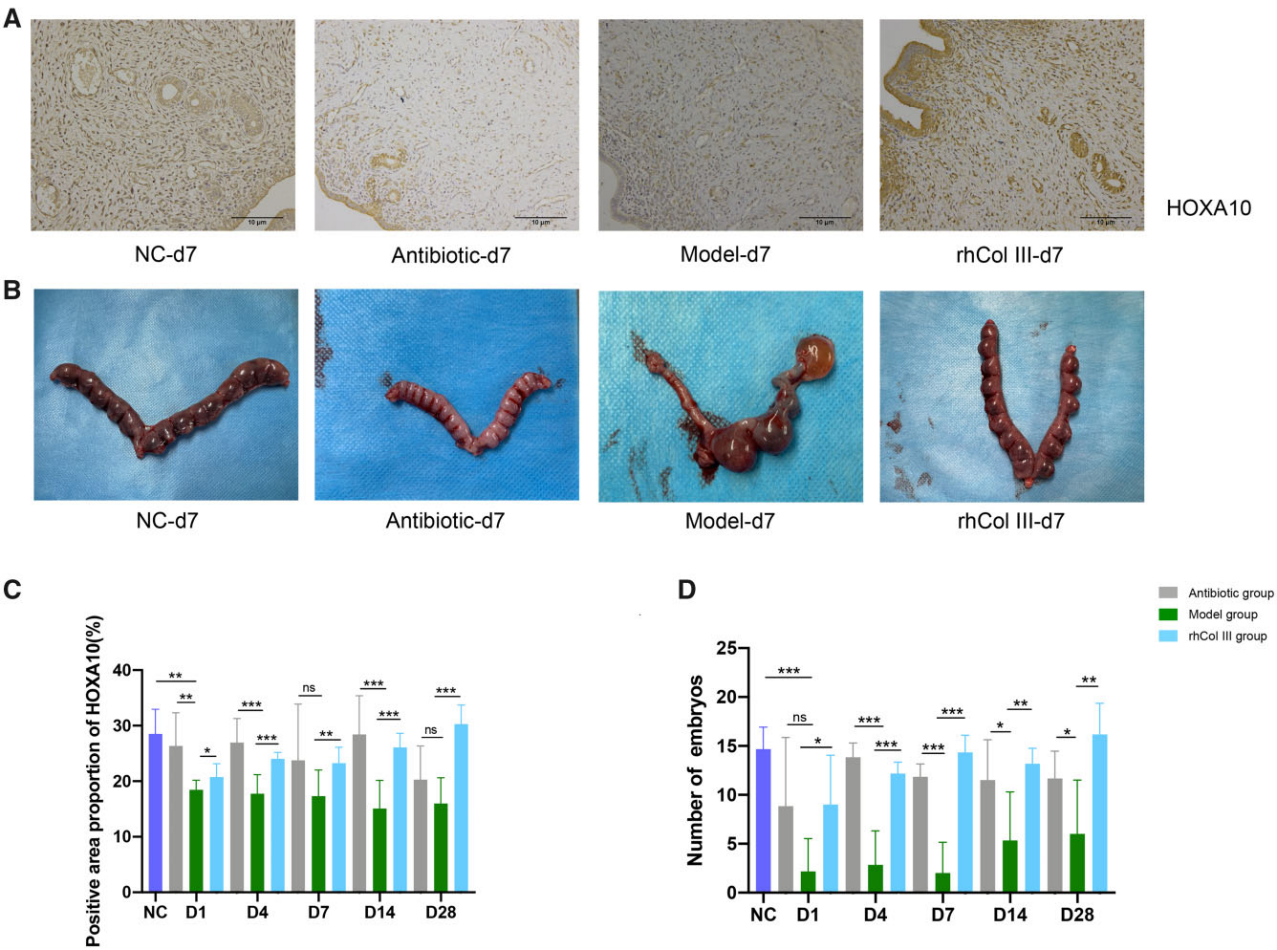
rhCol III promoted the expression of HOXA10 and the fertility of CE rats. (**A**) Immunohistochemical staining of HOXA10 of endometrium of four groups on Day 7 at 200× magnification. Scale bar = 100 μm. (**B**) Morphology of embryos at gestational day 13.5 in four groups. (**C**) Statistical analysis of HOXA10 expression. (**D**) Quantification of embryo numbers. (*P < 0.05, **P < 0.01, ***P < 0.001).

HOXA10 has been considered a marker to measure endometrium receptivity. The immunostaining of HOXA10 at Day 7 is shown in Fig. 8A and the others are seen in Supplementary Fig. S6B. HOXA10-positive signals were observed in the endometrium of all groups. When compared with the model group, the expression of HOXA10 in the rhCol III group was significantly increased throughout the observation period. In the antibiotic group, HOXA10 expression was higher than in the model group on Days 1, 4 and 14 (Fig. 8C). There was no significant difference at Days 7 and 28 between the antibiotic group and the model group.

Meanwhile, reproductive outcomes are also vital to assessing the efficacy of treatment. The embryo numbers were counted in our experiment. Embryos at Day 7 are shown in Fig. 8B, and embryos at Days 1, 4, 14 and 28 are seen in Supplementary Fig. S6C. As shown in Fig. 8D, there were very few embryos in the model group on Days 1 and 4 post-treatment, and a few embryos were observed on Days 7, 14 and 28. Both rhCol III and antibiotic treatments caused the number of embryos to increase compared with the model group (Fig. 8D). Those results were nearly consistent with the HOXA10 expression results.

## Discussion

**Figure 9.**
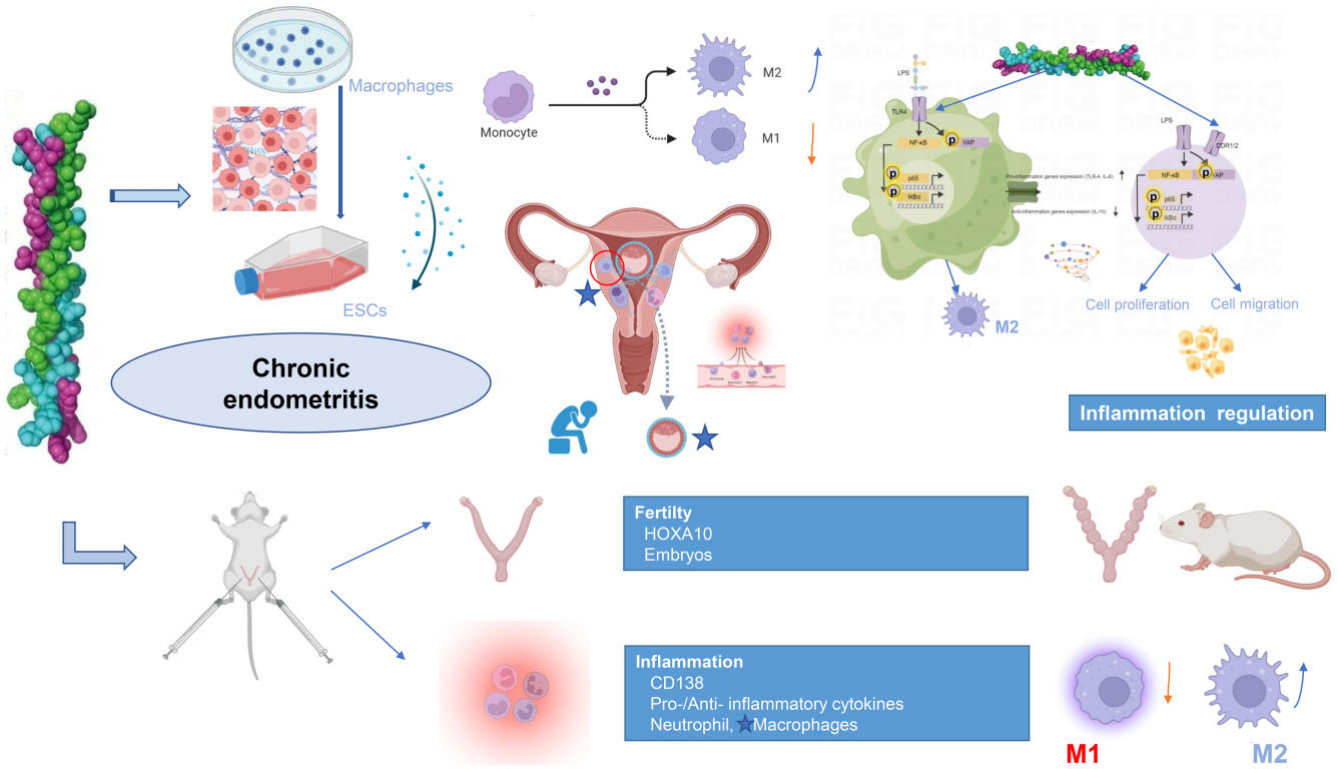
Schematic illustration of rhCol III for inflammation regulation and fertility restoration by immunomodulation in chronic endometritis.

Previous studies reported that CE has not been sufficiently researched due to its unspecific clinical manifestation, but it is closely related with implantation failure and unsatisfactory pregnancy outcomes [53–55]. Pathogen infection is considered the main cause of CE, although a study showed that some infertile CE patients, no pathogen was found in the endometrium but the immune microenvironment was still altered [56, 57]. Considering the primary cause of CE is infection, broad-spectrum antibiotic therapy is widely used to treat CE. However, the possible development of antibiotic resistance and the uncertainty surrounding the ability of antibiotics to improve pregnancy outcomes need to be taken into consideration [58–61]. The key points of CE treatment are to eliminate pathogens, regulate the immune microenvironment and promote endometrium regeneration for endometrial receptivity. Macrophages are closely connected with immune regulation and tissue regeneration, and they also play a vital role in endometrium regeneration during menstruation and endometrial receptivity establishment during successful pregnancy [15, 62–68]. It has been proved that macrophage polarization is essential to resistance against pathogen infection and to tissue repair [69, 70]. In CE patients, macrophage infiltration in the endometrium was related with microbiota imbalance and affected pregnancy outcome [71]. Therefore, mediated macrophage polarization could be a CE therapy strategy to promote anti-inflammatory cell infiltration and endometrium repair. Because rhCol III is a biomaterial with human type III collagen amino acid sequence, good biocompatibility and low immunogenicity, the biological effects of rhCol III on CE were evaluated (Fig. 9).

Due to the importance of macrophages in immune regulation and the CE pathological process, rhCol III promoted macrophage polarization to the M2 phenotype; this was observed in THP-1-differentiated M1 macrophages cultured in three different rhCol III concentrations. The addition of M1 macrophages cultured medium accelerated the production of pro-inflammatory cytokines and the reduction of anti-inflammatory factors of ESCs compared to LPS stimulation only. This indirectly and briefly explained the influence of macrophages on ESCs and the development of the inflammation microenvironment of the endometrium. The results of the *in vivo* investigation coincided with the results of the *in vitro* investigation. According to the histological results, macrophages participated in the inflammation process of CE, and rhCol III might have an effect on macrophages that works to alleviate the inflammation response. To investigate the potential mechanism involved in rhCol III’s effect on macrophages, the impact of rhCol III on macrophage polarization was observed. Every macrophage phenotype has a specific function. For instance, the M1 phenotype appears when the body is under infection and secretes pro-inflammatory factors to eliminate pathogenic microorganisms in the early immune response phase. In the later phase of the immune response, the M2 phenotype restrains the inflammatory response, expresses anti-inflammatory cytokines and takes part in tissue remodeling and regeneration. From the results *in vivo* and *in vitro*, the effect of rhCol III on immunomodulation mainly relies on macrophage polarization to the M2 phenotype. Furthermore, LPS, a kind of endotoxin, is produced by Gram-negative bacteria and could trigger inflammatory response mediated by macrophages and the secretion of inflammatory factors in body. Thus, LPS has been widely used to mimic inflammation models including endometritis [72]. Previous studies have illustrated that LPS-induced rat model is suitable for CE research [73]. In CE animal models, increased numbers of M1 macrophages were present in both the model and rhCol III groups within 4 days. From Day 7, the numbers of M1 macrophages in the rhCol III group decreased compared with the model group. For M2 macrophages, CD163^+^ cells were constantly at a lower level in the model group than in the rhCol III group, which suggested that rhCol III induced macrophage polarization to the M2 phenotype. Thus, the results of Day 7 were mainly demonstrated in figures as anti-inflammatory representative data. The expression of M1 macrophage-related pro-inflammatory cytokines (IL-6, TLR-4) was lower and the expression of M2 macrophage-related anti-inflammatory cytokines (IL-10) was higher in the rhCol III group than in the model group. The downregulation of the inflammatory response effectively provided a functional endometrium. In the antibiotic group, there were a few dynamic changes of macrophage-associated cells. This might illustrate that antibiotic therapy downregulates inflammatory cytokines rather than mediating macrophages. As a whole, rhCol III promoted macrophage polarization to the M2 phenotype to mediate the immune response and promote endometrial repair.

Subsequently, the biological functions of rhCol III on inflammatory ESCs were observed *in vitro*. The promotion of inflammatory ESCs accounted for the endometrial regeneration of CE. In addition, the ability of rhCol III to promote cell proliferation and migration was studied. Based on the high cell adhesion ability of rhCol III, the ECM–cell, cell–cell and cell behaviors could be promoted through cell adhesive interactions. Enhanced cell interactions were associated with the combination of collagen and its receptor on cell membranes. Because the repeat unit of rhCol III had the same amino acid sequence as human COL3A1 segment, it could interact with receptors such as integrins and DDRs. The DDRs are a subfamily of receptor tyrosine kinases widely expressed in tissues and are essential for the communication between cells and ECM. The DDRs include DDR1 and DDR2, are activated by collagen and are of significant importance to cell signaling, proliferation, migration and adhesion [74, 75]. DDR1 and DDR2 bind to collagen also to exert biological functions, like embryo development, ECM remodeling, organ fibrosis and osteoarthritis [76, 77]. According the results, the expression levels of DDR1, DDR2, collagen I and collagen III were increased after treatment with rhCol III, which supports the idea that rhCol III enhances DDR–collagen interactions and effectively promotes cell functions. Similarly, ECM remodeling occurred after treatment with rhCol III in CE animal models. The ECM is an important structural component of the endometrium, and it also regulates cell behaviors [78]. In the study of fibrotic diseases, macrophage–MMP–ECM interaction was involved in fibrosis and inflammation progression, and the restoration of ECM homeostasis could be beneficial for tissue microenvironment and regeneration. Targeted the dynamics between macrophage and ECM might be a new therapeutic strategy for inflammation regulation and repair [79, 80]. From our results, ECM components including collagen I and III were downregulated after LPS stimulation. The supplement of rhCol III promoted ECM production from Day 4 and dynamically remodeled ECM synthesis and degradation. And the time points of M1/M2 polarization and ECM remodeling were both in the mid-term after LPS stimulation. The expressions of TIMP-1 and MMP-2 showed that the ECM was degraded at the early phase of the immune response and ECM was synthesized at the mid and late phases of the immune response, which might prove that ECM remodeling is involved in immune regulation and endometrial regeneration.

In a previous study, endometrial microorganisms and metabolism changes associated with immune cell regulation were investigated in CE patients [72]. Thus, metabolism changes were examined to determine whether rhCol III treatment could mediate inflammation-related metabolites. The metabolism products of M1-cultured medium and LPS-stimulated inflammatory ESCs and three different concentrations of rhCol III co-cultured inflammatory ESCs were examined. From our results, rhCol III-treated inflammatory ESCs produced some anti-inflammatory metabolites. A component inhibiting the NF-κB pathway, 5-aminosalicylic acid, was produced in both the 10-rhCol III and 1000-rhCol III groups. In the 100-rhCol III group, the level of the well-known antibiotic amikacin showed a significant difference compared with the M1 + LPS-stimulated group. Previous research demonstrated that EPK could suppress macrophage-induced inflammation, and o-veratraldehyde was a midbody of antibiotics [81]. The differential metabolites proved rhCol III could promote the production of anti-inflammatory elements, which was consistent with our previous results. Interestingly, proline and its derivative glyclproline were present in the rhCol III groups. Proline is necessary for the biosynthesis of collagen [82]. The results might indicate that rhCol III could promote the production of collagen.

To explore the mechanism of how rhCol III affects immunomodulation, the function of rhCol III on macrophages and ESCs through the NF-κB/YAP signaling pathway was evaluated. NF-κB signaling is vital for immunological transcriptional programs, including innating immune cells for inflammatory responses to pathogens and viruses and proliferating and activating adaptive immune cells [83]. In a previous study, NF-κB p65 expression was higher in CE rats than in non-CE rats [84]. NF-κB is a transcription factor, which is composed of p65 and p50 subunits, and interacts with its inhibitory proteins IκBs (IκBα, β, ε, γ and δ). The phosphorylation of IκBα and p65 proteins are canonical markers of NF-κB activation [85, 86]. Primarily, M1 macrophages activated the expression of NF-κB, whereas rhCol III exerted a suppressive effect on NF-κB activation. IκB kinase (IKK) was first activated when stimulation signals were received by cellular receptors. Then, IKK phosphorylates caused the IκB subunits to ubiquitinate and degrade, which led to the release of NF-κB dimer (p50/p65). The expressions of p-65 and p-IκBα were inhibited in the rhCol III groups. To mimic the microenvironment *in vivo*, M1-cultured medium was used for LPS-induced ESCs. M1 medium-cultured ESCs exerted an enhanced inflammatory response, which included secreting more pro-inflammatory cytokines and activating the NF-κB signaling pathway. On inflammatory ESCs, rhCol III had a similar effect in that it suppressed the activation of p-65 and p-IκBα.

YAP, as a co-effector of the Hippo signaling pathway, plays a crucial role in cell proliferation and differentiation and ECM rigidity [87, 88]. YAP is involved in the inflammatory response and with NF-κB might regulate cell functions [89, 90]. A recent study showed that pro-inflammatory cytokine TNFα promoted interaction between p65 and YAP to synergistically regulate inflammation-driven migration [91]. At the same time, the latest studies have demonstrated that the Hippo signaling pathway is regulated in the uterus and affects uterine dynamic by regulating YAP and its targets [92, 93]. The function and phenotype of macrophages were modified by transcription factors (YAP, NF-κB) and YAP could mediate macrophages in lots of diseases [94–96]. According to the western blotting results, LPS and M1 macrophages cultured medium significantly increased the level of YAP-1 in the ESCs. However, rhCol III treatment increased YAP phosphorylation in both M1 macrophages and ESCs, and there were decreased YAP levels in the rhCol III group compared with the NC + 100LPS and NC + 100LPS + M1 groups. The stimulation of LPS induced NF-κB and YAP activation, which promoted inflammatory cytokines production in macrophages. To regulate macrophage polarization and achieve hemostasis were the key points of anti-inflammation. These data briefly indicated that rhCol III induces YAP phosphorylation by suppressing inflammation, and its inducement of YAP activation is correlated with NF-κB inhibition both in macrophages and ESCs. However, this research still had some limitations. A preliminary study above rhCol III regulated macrophage polarization and inflammation through NF-κB/YAP pathway, the precise mechanism of rhCol III’s effect on NF-κB and YAP interaction requires more in-depth research.

Finally, the increased expression of decidualization-related cytokines *in vitro* implied that rhCol III promotes the implantation process. *In vivo*, the endometrial receptivity and number of embryos are two crucial indexes to evaluate the treatment efficacy of CE directly and specifically. Under a 28-day observation period, endometrium infusion of rhCol III improved the inflammation response and promoted tissue regeneration to restore a normal endometrial environment. The CE rats presented lower embryo numbers, which might be caused by the constant inflammation stimulation and immune dysfunction of the endometrium. Although the rats conceived some embryos after Day 7, possibly proving that the endometrium had a certain self-regeneration ability, the embryo numbers were not at normal levels. In the rhCol III group, the embryo numbers were limited on Day 1 post-treatment. Over time, increasing numbers of embryos were conceived. HOXA10 is a marker of endometrium receptivity [97], and the result of HOXA10 expression in the endometrium is nearly consistent with pregnancy outcomes. Antibiotics were used as regular therapy for CE, and the improvements following rhCol III treatment were similar to those following antibiotic treatment. Overall, the results of this study suggested that rhCol III might promote restoration of a normal endometrium environment with suitable structural and functional conditions for embryo implantation and development.

## Conclusions

In summary, rhCol III is a biosynthesized material with good biocompatibility and effective biological functions. In this study, rhCol III alleviated the inflammatory response and promoted the restoration of a normal endometrial immune microenvironment via M2 macrophage polarization *in vitro* and *in vivo*. *In vitro*, rhCol III strengthened cell biological functions through collagen–DDR interactions and regulated cell metabolism. The mechanism of immunomodulation was associated with inhibition of the NF-κB/YAP signaling pathway. Heightened expression of decidualization-related cytokines, improved endometrial receptivity and increased embryo numbers effectively illustrated the fertility restoration and remodeled immune microenvironment after rhCol III treatment for CE. Therefore, rhCol III might be a promising biomaterial for regulating macrophages to provide CE therapy.

## Supplementary Material

rbad033_Supplementary_Data
