# Supplementary material for: Recombinant humanized collagen remodels endometrial immune microenvironment of chronic endometritis through macrophage immunomodulation
Source: Regen Biomater. 2023 Apr 3;10:rbad033. doi: 10.1093/rb/rbad033 (PMC10147517; doi:10.1093/rb/rbad033)
Supplement: rbad033_Supplementary_Data [file rbad033_supplementary_data.pdf]

Supplementary Information

Recombinant humanized collagen remodels endometrial immune microenvironment of chronic endometritis through macrophage immunomodulation

Shuang You [a, 1], Yun Zhu [e], Hu Li [a], Fan He [a, b], Shuaibin Liu [a], Xia Yang [f], Li Wang [a], Hui Zeng [a], Jingcong Dai [a], Lina Hu [a, b, c, d, *]

a Department of Obstetrics and Gynecology, The Second Affiliated Hospital, Chongqing Medical University, Chongqing 400010, China

b Joint International Research Lab for Reproduction and Development, Ministry of Education, Chongqing 400010, China

c Reproduction and Stem Cell Therapy Research Center of Chongqing, Chongqing 400010, China

d Center for Collagen Transformation of Chongqing Medical University, Chongqing 400010, China

e National Laboratory of Biomacromolecules, Institute of Biophysics, Chinese Academy of Sciences, Beijing 100101, China

f Shanxi Jinbo Pharmaceutical Co., Ltd., Taiyuan 030031, Shanxi, China

* Corresponding author.

* Correspondence to: L. Hu, Department of Obstetrics and Gynecology, The Second Affiliated Hospital, Chongqing Medical University, Chongqing 400010, China.

* Email address for correspondence: cqhulina@hospital.cqmu.edu.cn (L. Hu).

Supplemental tables

Table S1. Primary antibodies used in western blots.

Antibody	Company	Ratio
P65	Proteintech, China	1:1000
p-p65	CST, USA	1:1000
IκBα	Proteintech, China	1:1000
p-IκBα	CST, USA	1:1000
YAP-1	Proteintech, China	1:1000
p-YAP-1	CST, USA	1:1000
GAPDH	Proteintech, China	1:10000

Table S2. Sequence of q-PCR primers.

Primer	Sequence	
TLR4	F: CAGCTTTCGACAGTGAGGAGA	Human
	R: TTGTCGAGATGCTGCTGTGA	
	F: ACTTTATCCAGAGCCGTTGGTGTATC	Rat
	R: TCAAGGACAATGAAGATGATGCCAGAG	
IL-6	F: CACTGGTCTTTTGGAGTTTGAG	Human
	R: GGACTTTTGTACTCATCTGCAC	
	F: AGTTGCCTTCTTGGGACTGATGTTG	Rat
	R: GGTATCCTCTGTGAAGTCTCCTCTCC	
IL-10	F: GTTGTTAAAGGAGTCCTTGCTG	Human

	R: TTCACAGGGAAGAAATCGATGA	
	F: GGCAGTGGAGCAGGTGAAGAATG	Rat
	R: TGTCACGTAGGCTTCTATGCAGTTG	
Collagen I	F: GATTCCCTGGACCTAAAGGTGC	Human
	R: AGCCTCTCCATCTTTGCCAGCA	
	F: CCTCAGGGTATTGCTGGACAAC	Rat
	R: CAGAAGGACCTTGTTTGCCAGG	
Collagen III	F: TGGTCTGCAAGGAATGCCTGGA	Human
	R: TCTTTCCCTGGGACACCATCAG	
	F: GACCAAAAGGTGATGCTGGACAG	Rat
	R: CAAGACCTCGTGCTCCAGTTAG	
TIMP-1	F: CGAGACCACCTTATACCAGCGTTATG	Rat
	R: CGGTTCTGGGACTTGTGGACATATC	
MMP-2	F: CAAGGATGGACTCCTGGCACAT	Rat
	R: TACTCGCCATCAGCGTTCCCAT	
DDR1	F: GAGTGGATTTCCCTCGATCTC	Human
	R: GGGGAAATCAAGACTAACCAGA	
DDR2	F: AACGAGAGTGCCACCAATGGCT	Human
	R: ACTCACTGGCTTCAGAGCGGAA	
IGFBP-1	F: CTATGATGGCTCGAAGGCTC	Human
	R: TTCTTGTTGCAGTTTGGCAG	

PRL	F: TGACCCTTCGAGACCTGTTTG	Human
	R: CTTGCTCCTTGTCTTCGGG	
GAPDH	F: GTCTCCTCTGACTTCAACAGCG	Human
	R: ACCACCCTGTTGCTGTAGCCAA	
	F: GACATGCCGCCTGGAGAAAC	RAT
	R: AGCCCAGGATGCCCTTTAGT	

Supplemental figures

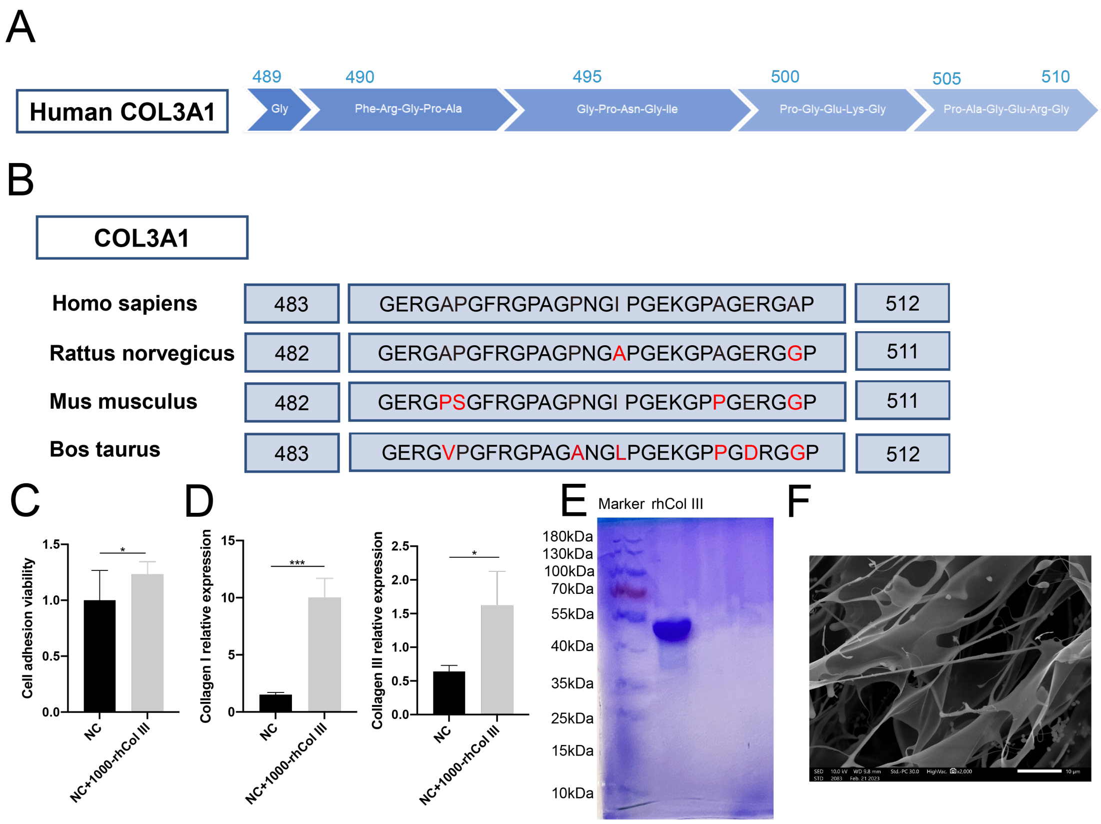

Fig. S1. rhCol III had specific sequence, structure and promote cell adhesion, collagen secretion. (A) The amino acid sequence of human COL3A1 (Gly489− Gly510). (B) Homology alignment of the COL3A1 sequence (483–512 segment) from Homo sapiens, Rattus norvegicus, Mus musculus, and Bos taurus. (C) The ESCs cell adhesion ability was detected after coating with rhCol III at 1000 μg/ml. (D) Statistical analysis of collagen I and III expressions of ESCs

and ESCs cultured with 1000 μg/ml rhCol III. (E) The molecular weight of rhCol III. (F) The morphology of collagen fiber was examined by SEM (scale bar = 10 μm) (*P < 0.05, **P < 0.01).

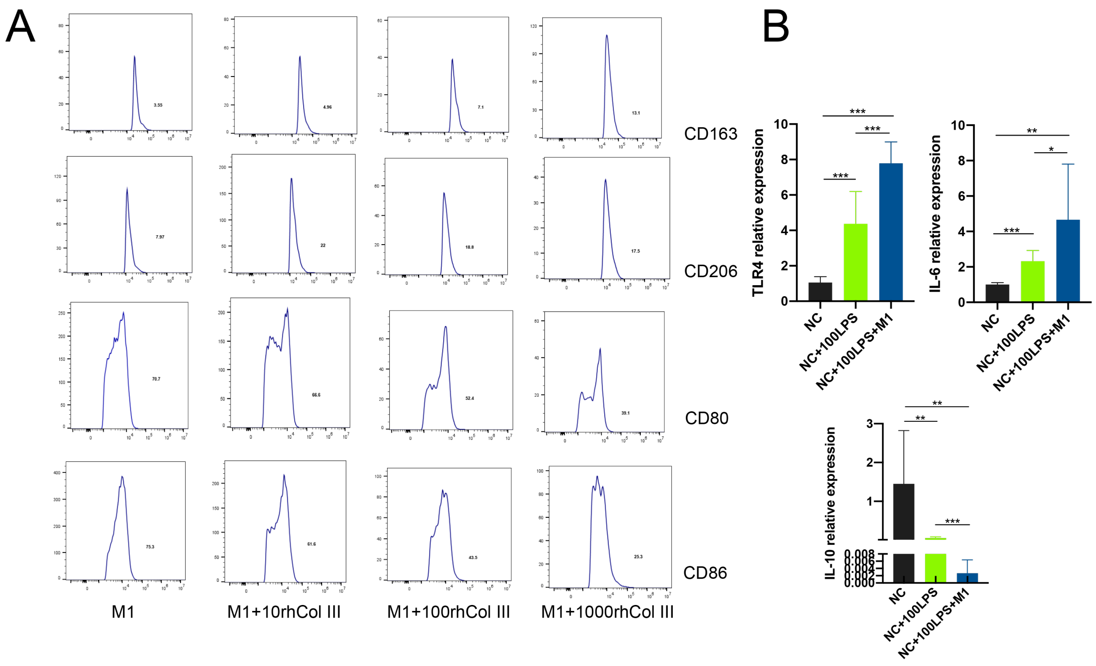

Fig. S2. rhCol III modulated macrophages polarization, pro-inflammatory and anti-inflammatory cytokines expression of ESCs. (A) The expressions of CD163, CD206, CD80 and CD86 of M1, 10-rhCol III, 100-rhCol III, 1000-rhCol III groups were detected by flow cytometry. (B) Statistical analysis of TLR-4, IL-6 and IL-10 expressions of NC, NC+100LPS, NC+100LPS+M1 groups (*P < 0.05, **P < 0.01).

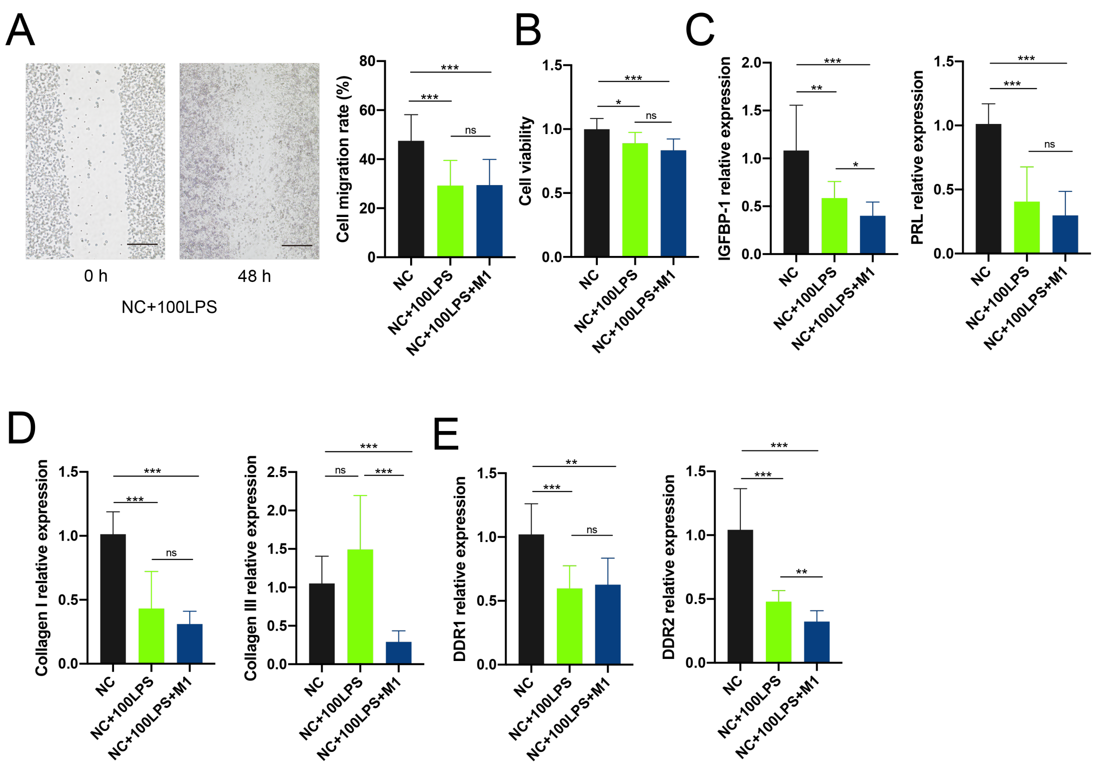

Fig. S3. The cell migration, viability and qualification analysis of ESCs. (A) Cell migration of NC+100LPS group during 48h, and statistical analysis of NC, NC+100LPS, NC+100LPS+M1 groups. (B) Statistical analysis of cell viability of NC, NC+100LPS, NC+100LPS+M1 groups. (C) Statistical analysis of IGFBP-1 and PRL expressions of NC, NC+100LPS, NC+100LPS+M1 groups. (D) Statistical analysis of collagen I and III expressions of NC, NC+100LPS, NC+100LPS+M1 groups. (E) Statistical analysis of DDR1 and DDR2 expressions of NC, NC+100LPS, NC+100LPS+M1 groups. (*P < 0.05, **P < 0.01).

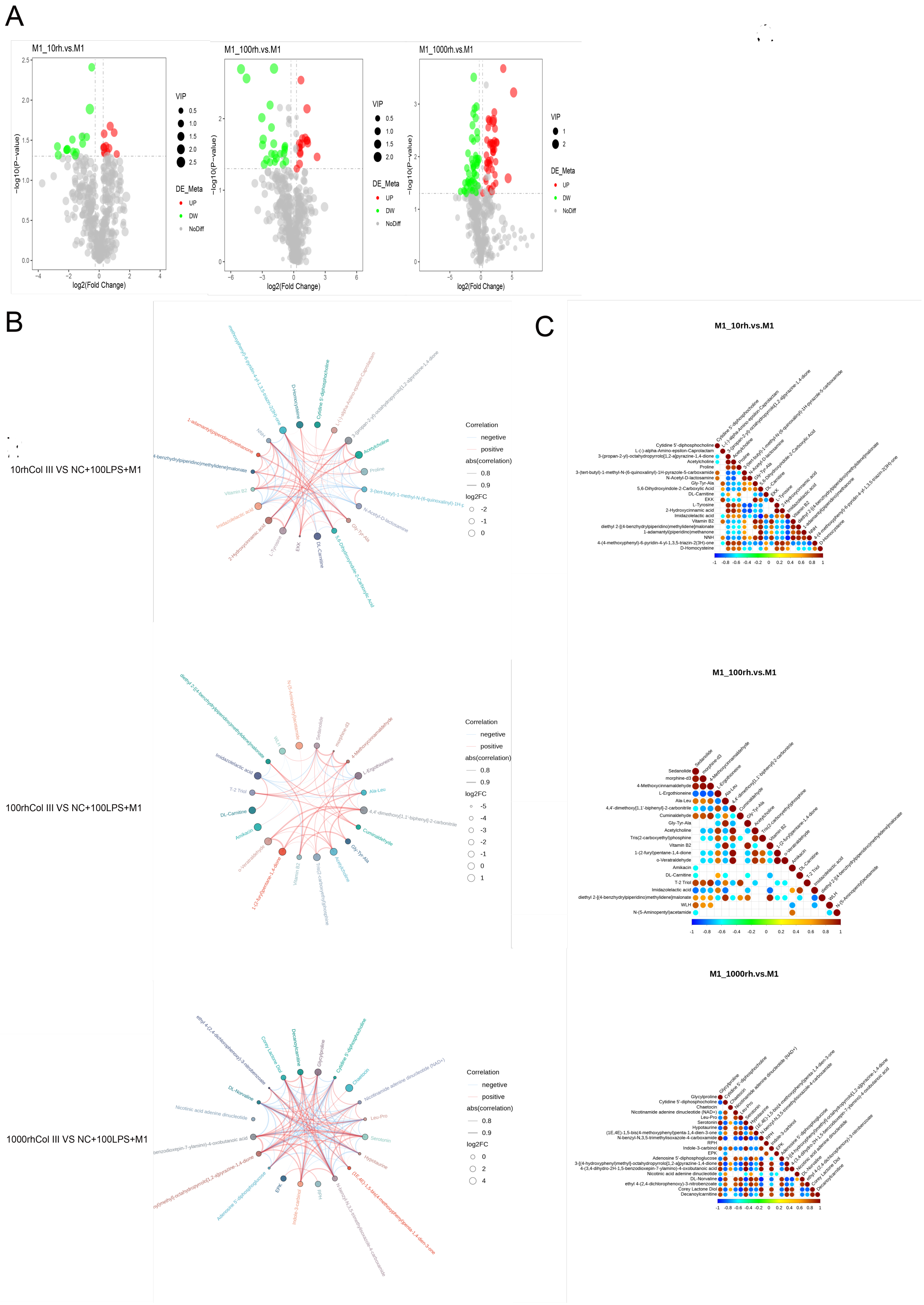

Fig. S4. Volcano maps and analysis of metabolites. (A) In 10-rhCol III, 100-rhCol III and 1000-rhCol III groups compared with NC+100LPS+ M1 group, 11, 16 and 46 metabolites were up-regulated and 14, 23 and 53 metabolites were down-regulated separately. (B) Chord diagrams of differential metabolites. (C) The correlation analysis of differential metabolites (*P < 0.05, **P < 0.01).

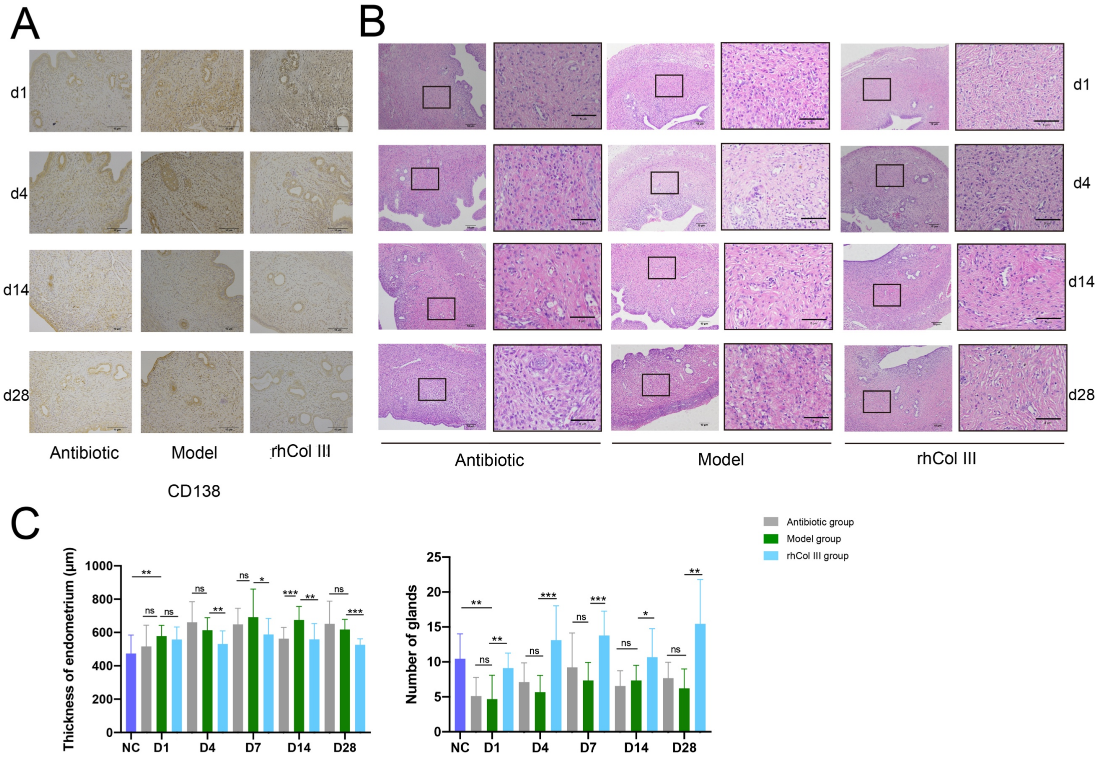

Fig. S5. The CD138 expression and histological illustration. (A) Immunohistochemical staining of CD138 in endometrium at day 1, 4, 14 and 28 at 200x magnification of antibiotic, model and rhCol III groups. Scale bar=10μm. (B) The H&E staining of endometrium of antibiotic, model and rhCol III groups at day 1, 4, 14 and 28 at 100x and 400x magnification Scale bar = 100 μm or 50 μm. (C) Statistical analysis of endometrium thickness and gland numbers of NC, antibiotic, model and rhCol III groups at day 1, 4, 14 and 28 (*$P < 0.05$, **$P < 0.01$).

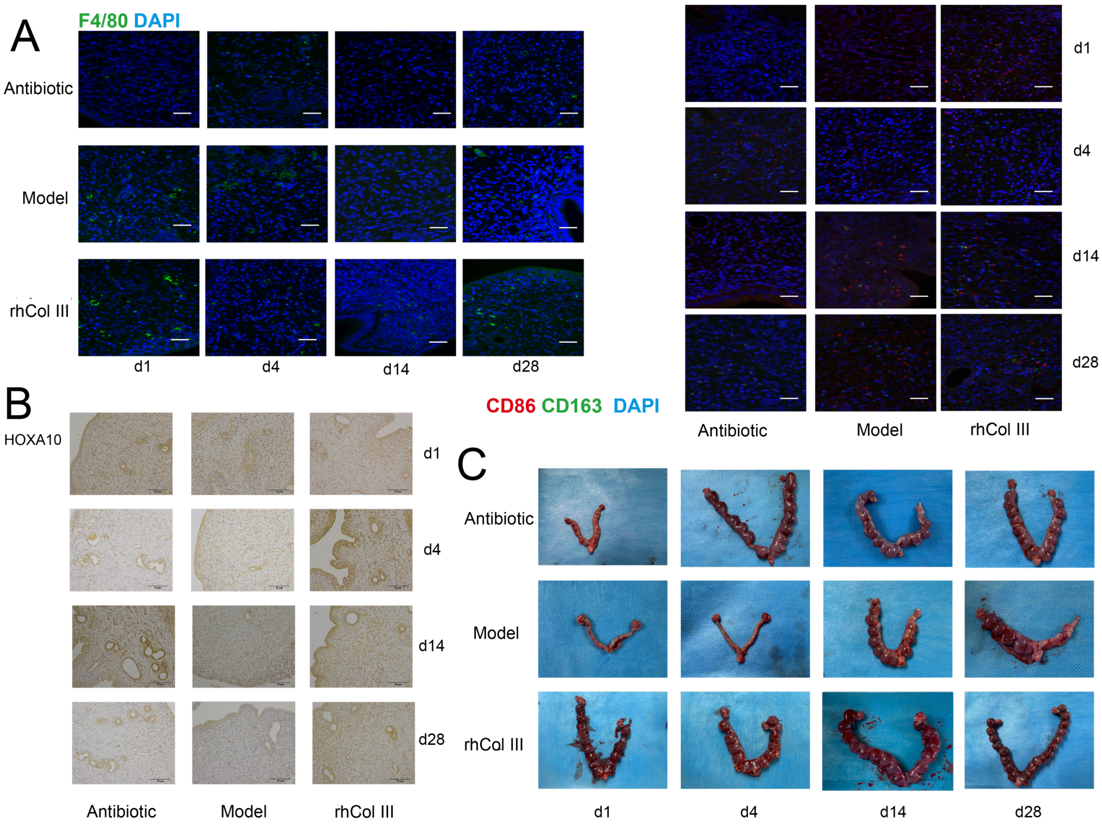

Fig. S6. Effects of rhCol III on macrophage regulation and pregnancy outcomes in vitro. (A) Immunofluorescence staining of F4/80, CD86 and CD163 of endometrium of antibiotic, model and rhCol III groups at day 1, 4, 14 and 28 at 200× magnification. Scale bar = 100 μm. The staining color of F4/80^{+} cells was green. The staining color of CD86^{+} cells was red and CD163^{+} cells were green. (B) Immunohistochemical staining of HOXA10 of endometrium of antibiotic, model and rhCol III groups at day 1, 4, 14 and 28 at 200× magnification. Scale bar = 100 μm. (C) Morphology of embryos at gestational day 13.5 of antibiotic, model and rhCol III groups.
